# A *m*-quaterphenyl probe for absolute configurational assignments of primary and secondary amines

**DOI:** 10.3762/bjoc.21.168

**Published:** 2025-10-20

**Authors:** Yuka Takeuchi, Mutsumi Kobayashi, Yuuka Gotoh, Mari Ikeda, Yoichi Habata, Tomohiko Shirai, Shunsuke Kuwahara

**Affiliations:** 1 Department of Chemistry, Faculty of Science Toho University, 2-2-1 Miyama, Funabashi, Chiba 274-8510, Japan; 2 Education Center, Faculty of Engineering, Chiba Institute of Technology, 2-1-1 Shibazono, Narashino, Chiba 275-0023, Japan; 3 Research Center for Materials with Integrated Properties, Toho University, 2-2-1 Miyama, Funabashi, Chiba 274-8510, Japan

**Keywords:** absolute configuration, chiral amine, chiral quaternary ammonium salt, circular dichroism, DFT calculation

## Abstract

We report a method for determining the absolute configurations of chiral amino alcohols, amino acid esters, and secondary amines through the combined use of a *m*-quaterphenyl probe **1** and theoretical calculations. The probe **1** is covalently attached to chiral amines to form conjugates that exhibit exciton-coupled circular dichroism (ECCD) in the *m*-quaterphenyl chromophores. The calculated ratios of the *P* and *M* conformers, obtained via DFT calculations, show a correlation with both the sign and intensity of the experimentally observed CD spectra.

## Introduction

Determining the absolute configurations of both natural and synthetic compounds continues to pose a considerable challenge in the life and materials sciences [1]. While X-ray crystallography remains a reliable method for this purpose, the requirement for high-quality single crystals often limits its applicability. In recent years, empirical approaches based on ^1^H NMR anisotropy method have gained attention as alternative strategies for stereochemical assignment of chiral molecules. Among these, the modified Mosher method, which utilizes the ring current effects of aryl substituents, has been extensively applied to chiral alcohols [2]. However, its use in the analysis of chiral amines has been restricted, largely due to the complexity arising from their conformational flexibility [3].

Circular dichroism (CD) spectroscopy offers a highly sensitive technique for stereochemical analysis at the microgram scale [4–6]. In particular, exciton-coupled CD has emerged as a powerful chiroptical method, providing a non-empirical correlation between the sign of the Cotton effect and the spatial arrangement of the electric transition dipole moments of interacting chromophores [7]. More recently, CD-based chiroptical probes have been developed for determining the absolute configurations of chiral alcohols [8–14], primary amines [14–32], secondary amines [33–35], carboxylic acids [36–38], sulfoxides [39], and cyanohydrins [40].

We have reported a *m*-quaterphenyl probe **1** to determine the absolute configurations of primary amines [41]. When compound **1** is linked to the amines, the information on the absolute configurations were transcribed into a twist of two biphenyl chromophores in the *m*-quarterphenyl group. From the sign of the Cotton effect in CD, the direction of twist can be estimated. The absolute configuration of amines can be determined by comparing the direction of the twist determined by CD with that obtained by conformational analysis using theoretical calculations. However, this method has only been applied to simple primary amines. In this work, we report that the method was applied to chiral amino alcohols and amino acid esters. We also applied the method to chiral secondary amines, for which it is generally difficult to determine the absolute configuration due to the conformational complexity of their derivatives [33]. By comparing the observed and calculated sign of the CD Cotton effect, their absolute configurations were determined.

## Results and Discussion

**Scheme 1:**
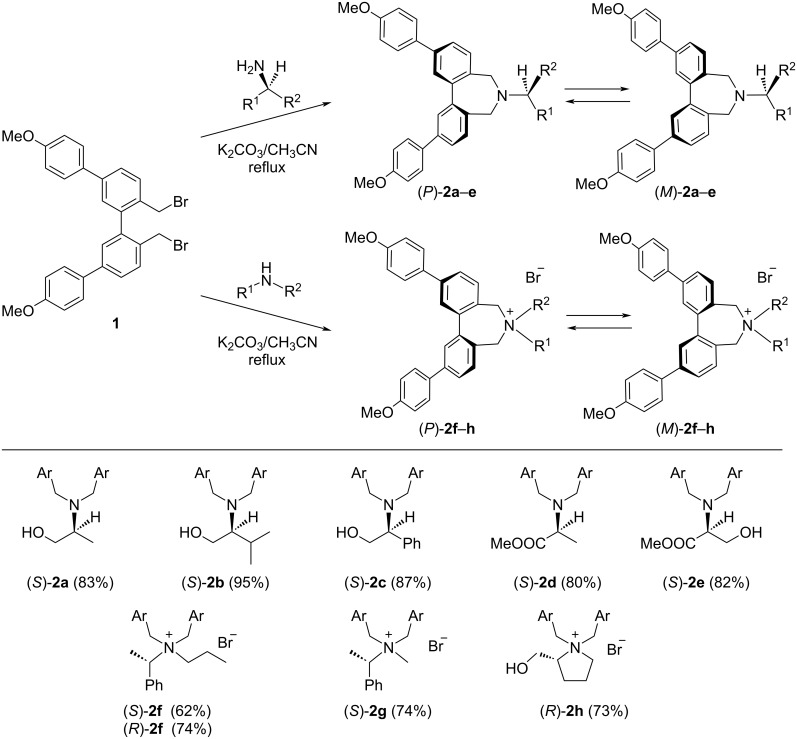
Coupling reaction of **1** and chiral primary and secondary amines. The central biphenyl moiety rotates freely, forming an equilibrium mixture of *P* and *M* conformers.

The probe **1** was prepared as described previously [41]. Probe **1**–primary amine conjugates **2a**–**e** were prepared by the reaction of **1** with chiral amino alcohols and amino acid esters in the presence of K_2_CO_3_ in CH_3_CN (Scheme 1). The quaternary ammonium salt conjugates **2f**–**h** were also prepared by reacting **1** with chiral secondary amines under similar conditions. It is reported that the central biphenyl moiety of amines and ammonium salts with seven-membered rings freely rotates at room temperature [41–43]. The central biphenyl moiety of conjugates **2a**–**h** also rotates freely, forming an equilibrium mixture of *P* and *M* conformers. The relative amounts of *P* and *M* conformers depend on the chirality of the linked amine moieties.

**Figure 1:**
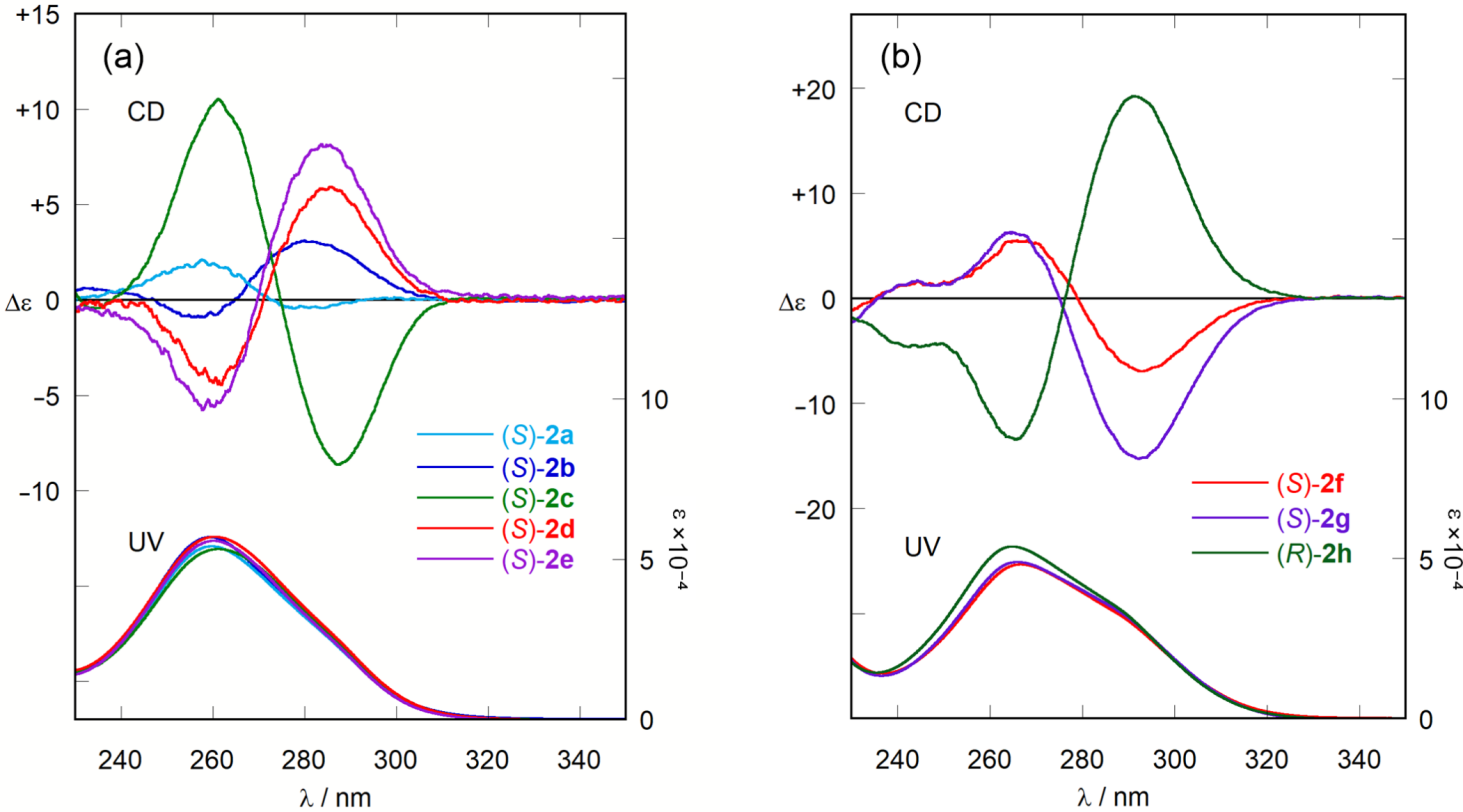
CD and UV spectra of (a) tertiary amines (*S*)-**2a**–**e** and (b) quaternary ammonium salts (*S*)-**2f**,**g** and (*R*)-**2h** (2.0 × 10^−4^ M in CH_3_CN, 293 K).

Figure 1a shows the UV and CD spectra of the conjugates (*S*)-**2a**–**e** in CH_3_CN. The UV and CD spectra of the previously reported conjugates were measured in various polar and non-polar solvents, but the intensities and shapes were almost unchanged [41]. The conjugates (*S*)-**2a**–**e** are composed of two methoxy-biphenyl chromophores connected with a C–C single bond. Therefore, the π-electron conjugation is widespread almost over the chromophores. However, the UV spectrum of **1**–ʟ-alaninol conjugate (*S*)-**2a** shows an intense absorption, maintaining the nature of methoxybiphenyl chromophore. The absorption band at 259 nm is attributed to the π–π* transition polarized along the long axes of the methoxybiphenyl chromophores. The CD spectrum of (*S*)-**2a** shows Cotton effects arising from exciton-coupling between the two methoxybiphenyl chromophores; λ_ext_ = 278 nm (Δε_1_ = −0.6 dm^3^ mol^−1^ cm^−1^) and λ_ext_ = 258 nm (Δε_2_ = +1.7 dm^3^ mol^−1^ cm^−1^). The amplitude of exciton-coupled CD (*A*_CD_ value) [7], defined as *A*_CD_ = Δε_1_ (first Cotton effect) − Δε_2_ (second Cotton effect)_,_ is measured to be −2.3 dm^3^ mol^−1^ cm^−1^.

The direction of the twist of the two chromophores in biaryl compounds can be determined by the sign of the Cotton effect of the exciton-coupled CD. Maison [44], Hanazaki [45], and Salvadori [46] have used chiral 1,1-binaphthyl derivatives to clarify the relationship between the direction of the twist of the two naphthyl chromophores and the sign of the exciton-coupled CD. In this case, the information on the dihedral angle between the two naphthyl chromophores is required [7]. When the angle is between 0 and 110 degrees, the direction of twist of the chromophores can be determined by the CD exciton method.

From the conformational analyses of the previously reported conjugates, the dihedral angle in the two methoxybiphenyl chromophores of (*S*)-**2a** is predicted to be approximately 42 degree. The negative first and positive second Cotton effects in CD spectrum of (*S*)-**2a** indicates that the two long axes in the methoxybiphenyl chromophores constitute an *M* twist. The CD spectra of **1**–ʟ-valinol conjugate (*S*)-**2b** exhibited the opposite sign for the Cotton effects compared with (*S*)-**2a**. This inversion of Cotton effects is due to the difference in steric features between the two substituents of amines: methyl and hydroxymethyl group in (*S*)-**2a**, and isopropyl and hydroxymethyl group in (*S*)-**2b**. The CD spectra of **1**–(*S*)-2-phenylglycinol conjugate (*S*)-**2c** showed the same sign for the Cotton effects compared with (*S*)-**2a**. We consider that the hydroxymethyl group is sterically more hindered than the planar phenyl groups in the conjugates. This tendency is the same as that of the previously reported **1**–(*S*)-2-phenylethylamine conjugates [41]. The CD spectra of **1**-ʟ-amino acid ester conjugates (*S*)-**2d**,**e** exhibited positive first and negative second Cotton effects indicating that the two long axes in the methoxybiphenyl chromophores constitute a *P* twist. The methyl ester group is estimated to be sterically more hindered than the planar ester carbonyl group in (*S*)-**2d**,**e**.

The CD spectra of the quaternary ammonium salts (*S*)-**2f**,**g** and (*R*)-**2h** exhibit CD Cotton effects due to the exciton-coupling between the two methoxy-biphenyl chromophores (Figure 1b). Compound (*R*)-**2f** exhibited the mirror image of the CD spectrum of (*S*)-**2f** (Figure S19 in Supporting Information File 1). The direction of twist of the two chromophores of quaternary ammonium salts (*S*)-**2f**,**g** and (*R*)-**2h** can be determined from the sign of the Cotton effect in CD. However, it is more difficult to predict the direction of twist from the structure of the quaternary ammonium salts than in the case of the **1**–primary amine conjugates.

**Figure 2:**
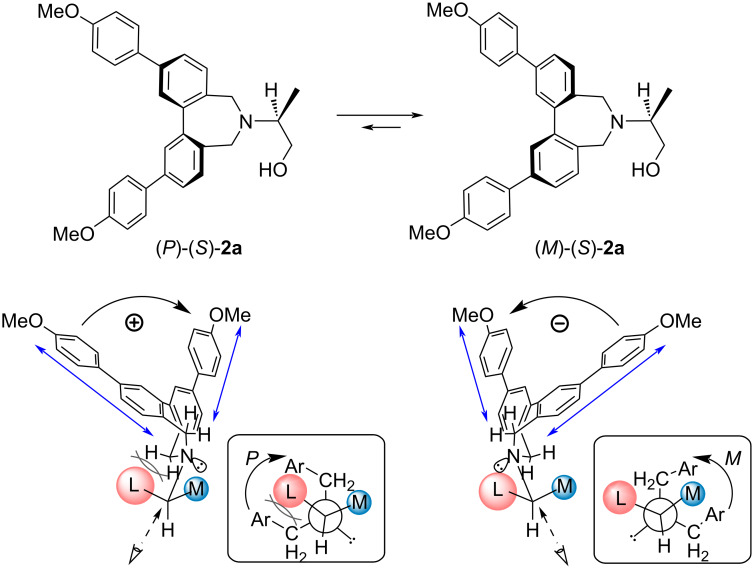
Schematic representation of the preferred conformation of (*S*)-**2a**.

Since the CD spectra revealed the twist between the two methoxybiphenyl chromophores, we next examined the relationship between the absolute configuration of the amine and the observed twist. Based on the previously reported stable conformations obtained from theoretical calculations of related derivatives [41] the preferred conformation of (*S*)-**2a** can be proposed (Figure 2). In the case of the *P* conformer, a Newman projection along the C–N bond reveals that the bulkier substituent, the hydroxymethyl group (denoted as L), is close to a seven-membered ring, and is destabilized by steric repulsion with the methylene protons. In contrast, in the *M* conformer, the medium-sized methyl group (denoted as M) is located near the seven-membered ring, reducing the steric repulsion involving the hydroxymethyl group with the methylene protons. Moreover, considering that the phenyl ring and the ester carbonyl group are planar, while the methyl group is more sterically demanding in three-dimensional space, this conformational model is supported (Table S1 in Supporting Information File 1). Although intramolecular electronic interactions should also be considered, the twist of the phenyl chromophores can be reasonably predicted by simply evaluating the relative steric bulkiness of substituents near the amine moiety. The relative substituents’ priority is determined not by the CIP rule, but by the steric bulkiness of substituents. On the other hand, for quaternary amines, it was difficult to assess the relative sizes of the substituents and to predict the direction of the twist. Therefore, we next employed conformational analysis using theoretical calculations to determine the twist of the methoxybiphenyl chromophores.

**Figure 3:**
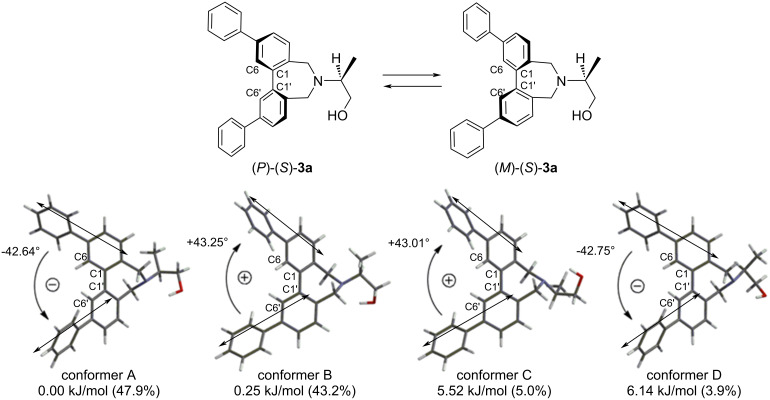
Four major conformers of (*S*)-**3a** based on B3LYP/6-31G* level.

**Figure 4:**
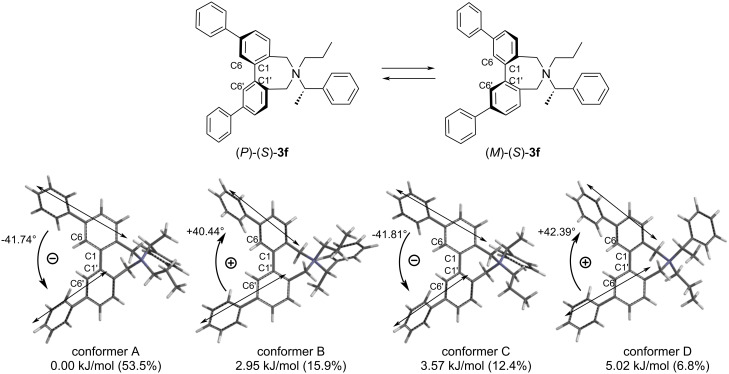
Four major conformers of (*S*)-**3f** based on B3LYP/6-31G* level.

To determine the direction of twist of the two methoxy-biphenyl chromophores of the conjugates (*S*)-**2a**–**g** and (*R*)-**2h**, theoretical calculations were carried out using a methoxy-omitted model (*S*)-**3a**–**g** and (*R*)-**3h**. To estimate the relative populations of the *P* and *M* conformers of (*S*)-**3a**, an initial conformational search was performed using the MMFF force field, followed by geometry optimizations of all local minima employing DFT at the B3LYP/6-31G* level of theory [47]. Four low-energy conformers of (*S*)-**3a** were identified within 10.0 kJ/mol (Figure 3). Among them, conformers A and D exhibited an *M* twist between the long axes of the biphenyl chromophores, whereas conformers B and C displayed a *P* twist. Based on a Boltzmann distribution analysis (*T* = 298 K), the relative populations of the *P* and *M* conformers in (*S*)-**3a** were determined to be 48:52. (Table S3 in Supporting Information File 1). In contrast, the *P* conformers were found to predominate in (*S*)-**3b** (Figure S21 and Table S4 in Supporting Information File 1). In the quaternary ammonium salts (*S*)-**3f**, the populations of the *M* conformers were greater than those of the *P* conformers (Figure 4). These computational results were consistent with the signs of the experimentally observed CD Cotton effects.

**Figure 5:**
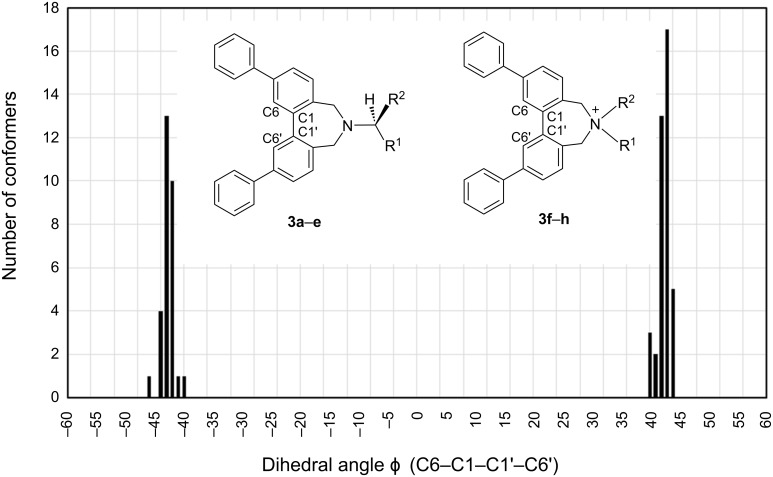
The distribution of conformers of (*S*)-**3a**–**h** against dihedral angles ϕ (C6–C1–C1'–C6') calculated by B3LYP/6-31G*.

**Figure 6:**
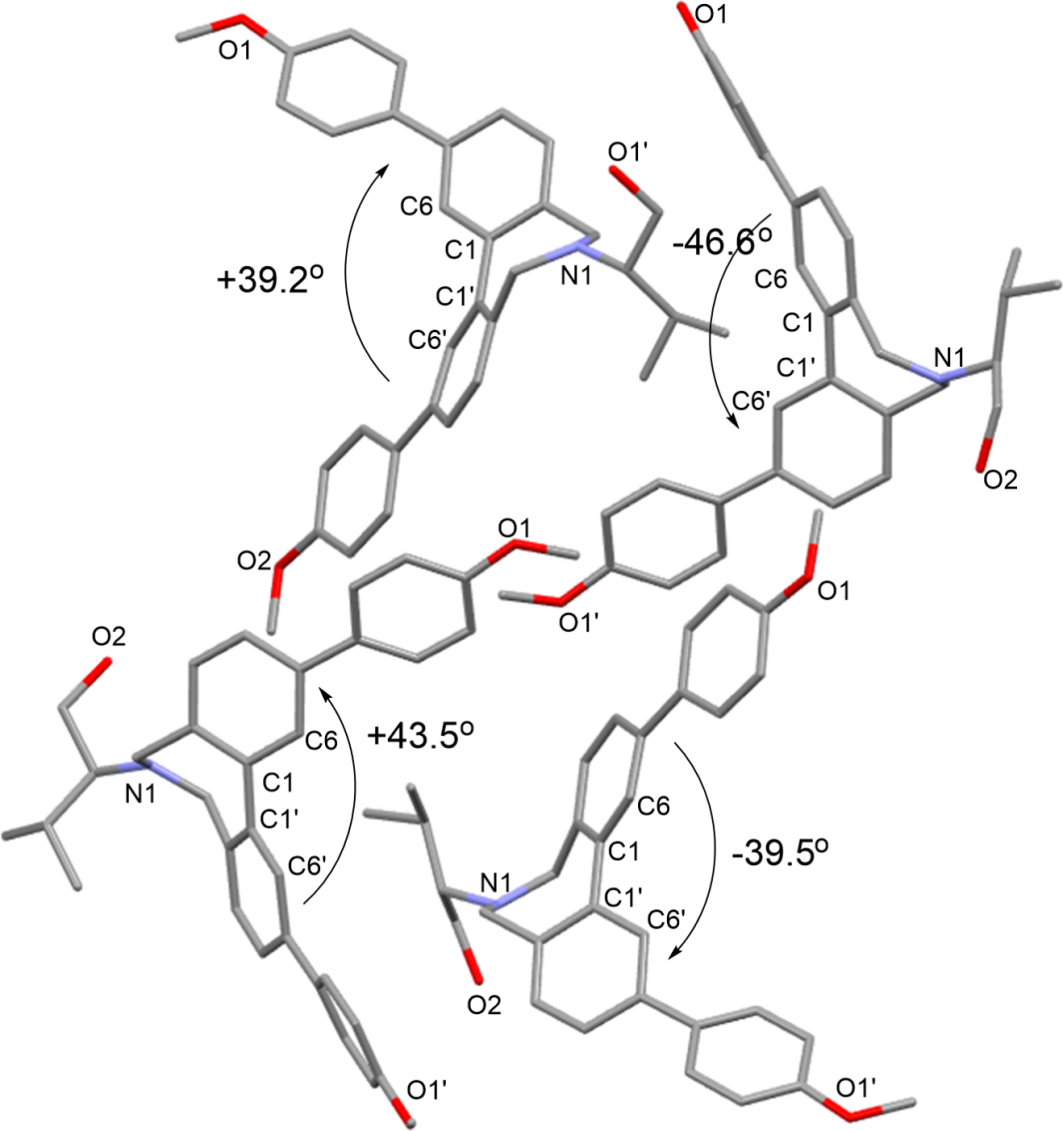
Crystal structure of (*S*)-**2b**. Four conformers exist in the unit cell. Hydrogen atoms are omitted for clarity.

From the conformational analyses described above, the C6–C1–C1'–C6' dihedral angles in all conformers of (*S*)-**3a**–**g** and (*R*)-**3h** were approximately constant (plus or minus ca. 42 degree). The range of distribution of the angles was also small, ranging from +39.8 to +43.9 degrees, or −40.2 to −45.8 degrees (Figure 5). In the solid state of (*S*)-**2b**, the C6–C1–C1'–C6' dihedral angles in the conformers were approximately constant. There are four conformers of (*S*)-**2b** in the unit cell, two of which are *P* and the other two are *M* conformers, with C6–C1–C1'–C6' dihedral angles of +39.2, +43.5, −39.5 and −46.6 degrees, respectively (Figure 6) [48].

**Figure 7:**
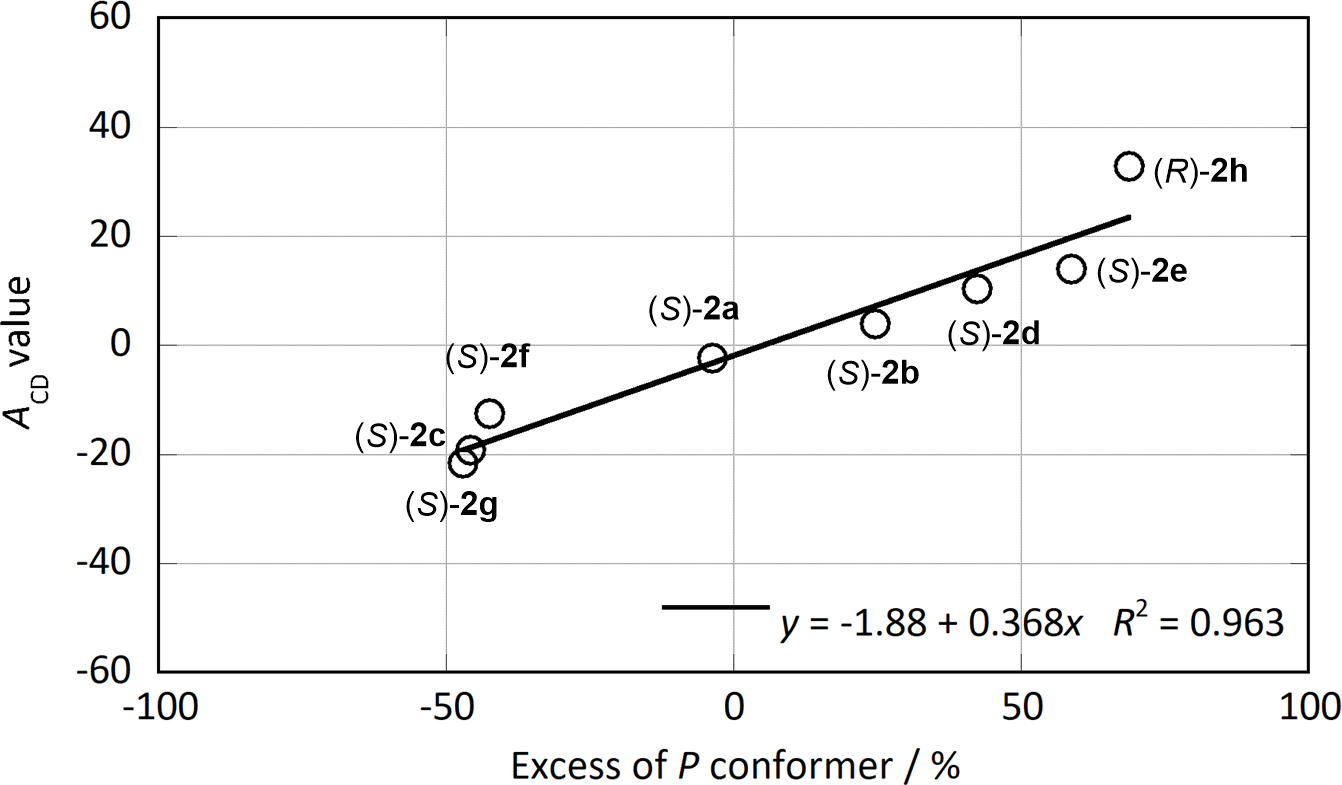
The relationship between the *A*_CD_ values and excess of *P* conformer. Excess of *P* conformer (%) = ([*P*] − [*M*])/([*P*] + [*M*]) × 100, where [*P*] and [*M*] are the amounts of *P* and *M* conformers calculated by B3LYP/6-31G*, respectively.

The intensity of the exciton-coupled CD depends on the torsion angle of the two chromophores [7]. Therefore, the CD intensity of (*S*)-**2a**–**g** and (*R*)-**2h**, directly reflects the abundance ratio of the *P* and *M* conformers. A linear relationship between the *A*_CD_ values and the calculated excess of *P* conformers, ([*P*] − [*M*])/([*P*] + [*M*]) × 100, was obtained with *R*^2^ = 0.963 (Figure 7). A similar linear correlation was observed when previously reported conjugates were applied (Figure S28 in Supporting Information File 1). By comparing the observed and calculated sign of the CD Cotton effect, the absolute configurations of chiral amines were determined. Reported chiroptical probes for determining the absolute configuration of chiral compounds only used the sign of the Cotton effect in CD. This method uses not only the sign but also the intensity of the Cotton effect in CD to determine the absolute configuration of chiral primary and secondary amines. In other words, by comparing the sign and the intensity of the CD using the relationship in Figure 7, the determination of the absolute configuration can be guaranteed.

## Conclusion

The combination of the CD spectra and conformational analysis by theoretical calculations using *m*-quaterphenyl probe **1** represents an effective method to determine both the absolute configuration of chiral primary amino alcohols and amino acid esters. This method is also useful for chiral secondary amines, for which it is generally difficult to determine the absolute configuration. By comparing the intensities of the *A*_CD_ values, the reliability of determining the absolute configurations can be guaranteed. Further application of the method to amines with more complex structures is currently in progress.

## Experimental

### General methods

All reagents and solvents were commercially available and used without further purification. Melting points were obtained with a Mel-Temp capillary apparatus and were not corrected. IR spectra were obtained as KBr disks on a JASCO FT/IR-410 spectrophotometer. The FAB mass spectra were recorded using a JEOL 600H mass spectrometer. ^1^H and ^13^C{^1^H} NMR spectra were recorded on a Jeol ECP400 spectrometer (400 MHz) and a Bruker AVANCE II spectrometer (400 MHz). All NMR spectroscopic data of CDCl_3_ solutions are reported in ppm (δ) downfield from TMS. UV and CD spectra were recorded on JASCO V-650 and JASCO J-820 spectrometers, respectively. X-ray single-crystal structure analysis was performed on a Bruker SMART diffractometer equipped with a CCD area detector at 120 K. Silica gel 60 F_254_ precoated plates on glass from Merck Ltd. were used for thin-layer chromatography (TLC).

### General procedure for the synthesis of conjugates **2a–h**

**(*S*)-2-[2,10-Bis(4-methoxyphenyl)-5,7-dihydro-6*H*-dibenzo[*c,e*]azepin-6-yl]propan-1-ol ((*S*)-2a).** A mixture of *m*-quaterphenyl probe **1** (290 mg, 0.526 mmol), ʟ-alaninol (48.3 mg, 0.643 mmol) and K_2_CO_3_ (294 mg, 2.12 mmol) in CH_3_CN (12 mL) was stirred at 85 °C for 3 h. After cooling to room temperature, the mixture was filtered through a pad of Celite, and then evaporated to dryness. The crude product was purified by column chromatography on silica gel (EtOAc) to yield amine (*S*)-**2a** (203 mg, 83% yield) as colorless solid: mp 164.2–165.5 °C; ^1^H NMR (400 MHz, CDCl_3_) δ 7.73 (d, *J* = 1.8 Hz, 2H), 7.62–7.56 (m, 6H), 7.44 (d, *J* = 7.8 Hz, 2H), 7.00 (dt, *J*_1_ = 8.7 Hz, *J*_2_ = 3.6 Hz, 4H), 3.86 (s, 6H), 3.68–3.46 (m, 6H), 3.20–3.12 (m, 2H), 1.12 (d, *J* = 6.6 Hz); ^13^C NMR (100 MHz, CDCl_3_) δ 159.3, 141.4, 140.7, 134.0, 133.2, 130.3, 128.2, 126.3, 126.0, 114.3, 63.4, 60.7, 55.4, 51.3, 13.3; IR (KBr) ν_max_: 3407, 2959, 1732, 1607, 1517, 1489, 1249, 1178, 1038, 822 cm^−1^; FABMS (matrix DTT/TG = 1:1) *m/z*: 465 [M]^+^ (100%); Anal. calcd for C_31_H_31_NO_3_: C, 79.97; H, 6.71; N, 3.01; found: C, 79.73; H, 6.75; N, 2.98.

### Theoretical calculations

To obtain the population between *M* and *P* conformers, preliminary conformational searches were run on the structures of (*S*)-**3a**–**g** and (*R*)-**3h** using MMFF. All local minimum conformers were then optimized with DFT using the B3LYP/6-31G* model [47]. The lower energy conformers with relative energies ranging from 0.0 to 10.0 kJ/mol were selected. By the Bolzmann distribution based on the energy difference of the conformers at 293 K, the population of the *P* and *M* conformers were determined.

## Supporting Information

File 1 Experimental procedures, characterization data including copies of NMR spectra (^1^H NMR, ^13^C NMR), CD spectra of (*S*)-**2f** and (*R*)-**2f**, theoretical calculations, and X-ray structure of (*S*)-**2b**.

File 2 Crystallographic information file for compound **2b**.

## Data Availability

All data that supports the findings of this study is available in the published article and/or the supporting information of this article.
