# Supplementary material for: A m-quaterphenyl probe for absolute configurational assignments of primary and secondary amines
Source: Beilstein J Org Chem. 2025 Oct 20;21:2211–9. doi: 10.3762/bjoc.21.168 (PMC12557418; doi:10.3762/bjoc.21.168)
Supplement: File 1 — Experimental procedures, characterization data including copies of NMR spectra (1H NMR, 13C NMR), CD spectra of (S)-2f and (R)-2f, theoretical calculations, and X-ray structure of (S)-2b. [file Beilstein_J_Org_Chem-21-2211-s001.pdf]

BEILSTEIN JOURNAL OF ORGANIC CHEMISTRY

Supporting Information

for

A *m*-quaterphenyl probe for absolute configurational assignments of primary and secondary amines

Yuka Takeuchi, Mutsumi Kobayashi, Yuuka Gotoh, Mari Ikeda, Yoichi Habata, Tomohiko Shirai and Shunsuke Kuwahara

Beilstein J. Org. Chem. **2025,** *21,* 2211–2219. doi:10.3762/bjoc.21.168

Experimental procedures, characterization data including copies of NMR spectra (^{1}H NMR, ^{13}C NMR), CD spectra of (*S*)-2f and (*R*)-2f, theoretical calculations, and X-ray structure of (*S*)-2b

License and Terms: This is a supporting information file under the terms of the Creative Commons Attribution License (https://creativecommons.org/licenses/by/4.0). Please note that the reuse, redistribution and reproduction in particular requires that the author(s) and source are credited and that individual graphics may be subject to special legal provisions.
The license is subject to the *Beilstein Journal of Organic Chemistry* terms and conditions: (https://www.beilstein-journals.org/bjoc/terms)

Table of contents

1. Synthesis of conjugates (*S*)-**2b**–**g**, (*R*)-**2f**, and (*R*)-**2h** S2

2. ^{1}H and ^{13}C NMR spectra of (*S*)-**2a**–**g**, (*R*)-**2f**, and (*R*)-**2h** S5

3. CD spectral data of (*S*)-**2a**–**e**,**i**–**n** S14

4. CD spectral data of (*S*)-**2f**–**h** S15

5. UV and CD spectra of (*S*)-**2f** and (*R*)-**2f** S15

5. Theoretical calculations of (*S*)-**3a**–**g** and (*R*)-**3h** S16

6. X-ray structure determination of (*S*)-**2b** S25

7. References S28

Synthesis of conjugates (*S*)-2b–g, (*R*)-2f, and (*R*)-2h

(*S*)-2-[2,10-Bis(4-methoxyphenyl)-5,7-dihydro-6*H*-dibenzo[*c,e*]azepin-6-yl]-3-methylbutan-1-ol ((*S*)-2b)

Following general procedure was carried out with **1** (70.3 mg, 0.127 mmol), L-valinol (26.1 mg, 0.253 mmol) and K_2CO_3 (118 mg, 0.850 mmol) in CH_3CN (4 mL) at 85 °C for 4 h. Purification using column chromatography (hexane/EtOAc, v/v 1:1) afforded amine (*S*)-**2b** (59.6 mg, 95% yield) as colorless prisms: mp 148.1–149.1 °C; ^{1}H NMR (400 MHz, $CDCl_3$) δ 7.74 (d, J = 1.8 Hz, 2H), 7.61–7.56 (m, 6H), 7.41 (d, J = 7.8 Hz, 2H), 6.99 (dt, J_1 = 8.7 Hz, J_2 = 3.8 Hz, 4H), 3.85 (s, 6H), 3.75–3.68 (m, 5H), 3.48 (t, J = 9.4 Hz, 4H), 2.67 (ddd, J_1 = 9.4 Hz, J_2 = 5.1 Hz, J_3 = 3.8 Hz, 1H), 2.01 (sep.d, J_1 = 6.9 Hz, J_2 = 5.1 Hz, 1H), 0.93 (dd, J_1 = 6.9 Hz, J_2 = 3.1 Hz, 6H); ^{13}C NMR (100 MHz, $CDCl_3$) δ 159.3, 141.3, 140.6, 134.2, 133.2, 130.1, 128.2, 126.4, 126.1, 114.3, 71.9, 59.8, 55.4, 52.1. 29.0, 23.0, 19.5; IR (KBr) ν_{max} = 3385, 2952, 2834, 1607, 1518, 1489, 1462, 1247, 1180, 1037, 1013, 820 cm^{-1}; FAB-MS (matrix DTT/TG = 1:1) *m/z* 494 $[M]^+$ 100%; Anal. Calcd for $C_{33}H_{35}NO_3$: C, 80.29; H, 7.15; N, 2.84. Found. C, 80.03; H, 7.09; N, 2.86.

(*S*)-2-[2,10-Bis(4-methoxyphenyl)-5,7-dihydro-6*H*-dibenzo[*c,e*]azepin-6-yl]-2-phenylethanol ((*S*)-2c)

Following general procedure was carried out with **1** (71.9 mg, 0.130 mmol), (*S*)-2-phenylglycinol (24.0 mg, 0.175 mmol) and K_2CO_3 (98.7 mg, 0.714 mmol) in CH_3CN (4 mL) at 85 °C for 3 h. Purification using column chromatography (hexane/EtOAc, v/v 2:1) afforded amine (*S*)-**2c** (58.9 mg, 87% yield) as colorless solid: mp 178.0–179.5 °C; ^{1}H NMR (400 MHz, $CDCl_3$) δ 7.72 (d, J = 1.8 Hz, 2H), 7.58 (dt, J_1 = 8.8 Hz, J_2 = 4.0 Hz, 4H), 7.53–7.32 (m, 7H), 7.26–7.25 (m, 2H), 6.99 (dt, J_1 = 8.8 Hz, J_2 = 4.0 Hz, 4H), 4.03 (dd, J_1 = 11.1 Hz, J_2 = 4.8 Hz, 1H), 3.93 (dd, J_1 = 11.1 Hz, J_2 = 4.8 Hz, 1H), 3.85 (s, 6H), 3.76 (t, J = 4.8 Hz, 1H), 3.66 (d, J = 12.4 Hz, 1H), 3.47 (d, J = 12.4 Hz, 1H), 2.27 (br, 1H); ^{13}C NMR (100 MHz, $CDCl_3$) δ 159.3, 141.6, 140.7, 133.3, 133.2, 130.3, 128.8, 128.7, 128.2, 127.9, 126.0, 125.8, 114.3, 68.5, 64.0, 55.4, 52.6; IR (KBr) ν_{max} = 3417, 3025, 2931, 2834, 1608, 1517, 1489. 1454, 1248, 1178, 1029, 820 cm^{-1}; FAB-MS (matrix DTT/TG = 1:1) *m/z* 529 $[M+1]^+$ 100%; Anal. Calcd for $C_{36}H_{33}NO_3$: C, 81.95; H, 6.30; N, 2.65. Found. C, 81.70; H, 6.33; N, 2.60.

Methyl (*S*)-2-(2,10-bis(4-methoxyphenyl)-5,7-dihydro-6*H*-dibenzo[*c,e*]azepin-6-yl)propanoate ((*S*)-2d)

Following general procedure was carried out with **1** (100.0 mg, 0.182 mmol), L-alanine methyl ester hydrochloride (28.3 mg, 0.203 mmol) and K_2CO_3 (150.0 mg, 1.09 mmol) in CH_3CN (6 mL) at 60 °C for 19 h. Purification using column chromatography (hexane/EtOAc, v/v 1:1) afforded amine (*S*)-**2d** (71.4 mg, 80% yield) as colorless solid: mp 62.9 °C (dec.); ^{1}H NMR (400 MHz, $CDCl_3$) δ 7.73 (d, J = 1.8 Hz, 2H), 7.62–7.55 (m, 6H), 7.43 (d, J = 7.9 Hz, 2H), 7.00 (dt, J_1 = 8.8 Hz, J_2 = 3.0 Hz, 4H), 3.86 (s, 6H), 3.74 (s, 3H), 3.68–3.54 (m, 5H), 1.50 (d, J = 6.9 Hz); ^{13}C NMR (100 MHz, $CDCl_3$) δ 174.4, 159.3, 141.5, 140.7, 133.3, 132.8, 130.6, 128.2, 126.0, 125.9, 114.3, 61.6, 55.4, 52.6, 51.9, 16.8; IR (KBr) ν_{max} = 3024, 2950, 1730, 1608, 1517, 1489, 1248, 1178, 1029, 821 cm^{-1}; FAB-MS (matrix: DTT:TG = 1:1) *m/z* 493($[M]^+$ 55%), Anal. Calcd. for $C_{32}H_{31}NO_4\cdot0.3H_2O$: C, 77.02; H, 6.38; N, 2.81. Found: C, 76.93; H, 6.09; N, 2.82.

Methyl (*S*)-2-(2,10-bis(4-methoxyphenyl)-5,7-dihydro-6*H*-dibenzo[*c,e*]azepin-6-yl)-3-hydroxypropanoate ((*S*)-2e)

Following general procedure was carried out with **1** (101.0 mg, 0.182 mmol), L-serine methyl ester hydrochloride (32.1 mg, 0.206 mmol) and K_2CO_3 (150.0 mg, 1.09 mmol) in CH_3CN (6 mL) at 50 °C for 18 h. Purification using column chromatography (EtOAc) afforded amine (*S*)-**2e** (76.3 mg, 82% yield) as colorless solid: mp 110.0 °C (dec.); ^{1}H NMR (400 MHz, $CDCl_3$) δ 7.72 (d, J = 1.8 Hz, 2H), 7.61–7.56 (m, 6H), 7.39 (d, J = 7.8 Hz, 2H), 7.00 (dt, J_1 = 8.7 Hz, J_2 = 3.1 Hz, 4H), 3.97–3.88 (m, 2H), 3.86 (s, 6H), 3.69 (s, 4H), 3.62–3.59 (dd, J_1 = 5.9 Hz, J_2 = 5.9 Hz, 1H), 3.56 (s, 3H); ^{13}C NMR (100 MHz, $CDCl_3$) δ 172.2, 159.3, 141.3, 140.8, 133.2, 132.9, 130.5, 128.2, 126.1, 126.0, 114.3, 66.5, 59.5, 55.4, 52.3, 51.5; IR (KBr) ν_{max} = 3423, 3025, 2951, 1730, 1608, 15178, 1490, 1248, 1178, 1038, 821 cm^{-1}; FAB-MS (matrix: *m*-NBA) *m/z* 509 ([M]$^+$ 68%), Anal. Calcd. for $C_{32}H_{31}NO_5 \cdot 0.12CHCl_3$: C, 73.63; H, 5.99; N, 2.67. Found: C, 73.49; H, 5.80; N, 2.66.

(*S*)-2,10-Bis(4-methoxyphenyl)-6-(1-phenylethyl)-6-propyl-6,7-dihydro-5*H*-dibenzo[*c,e*]azepin-6-ium bromide ((*S*)-2f)

Following general procedure was carried out with **1** (70.0 mg, 0.127 mmol), (*S*)-*N*-(1-phenylethyl)propan-1-amine [1] (23.1 mg, 0.142 mmol) and K_2CO_3 (110 mg, 0.799 mmol) in CH_3CN (4 mL) at 85 °C for 23 h. Purification using column chromatography ($CHCl_3$/MeOH, v/v 5:1) afforded ammonium salt (*S*)-**2f** (50.0 mg, 62% yield) as pale yellow solid: m.p. 125.1–126.1 °C; ^{1}H NMR (400 MHz, $CDCl_3$) δ 8.01 (d, J = 1.8 Hz, 1H), 7.97 (d, J = 1.8 Hz, 1H), 7.82–7.70 (m, 7H), 7.61 (dd, J_1 = 7.9 Hz, J_2 = 1.8 Hz, 1H), 7.95–7.34 (m, 4H), 7.08–7.02 (m, 4H), 5.12 (q, J = 7.0 Hz, 1H), 4.61 (dd, J_1 = 13.0 Hz, J_2 =13.0 Hz, 2H), 4.16 (dd, J_1 = 13.0 Hz, J_2 = 13.0 Hz, 2H), 3.82 (s, 6H), 3.59–3.49 (m, 1H), 3.27–3.14 (m, 1H), 2.04–1.90 (m, 5H), 0.90 (t, J = 7.3 Hz); ^{13}C NMR (100 MHz, $CDCl_3$) δ 159.8$_2$, 159.8$_0$, 142.4, 142.1, 141.0$_5$, 140.9, 134.2, 133.2, 132.8, 131.5, 131.0, 130.2, 129.0, 128.5, 128.3, 128.2$_4$, 128.2$_1$, 127.1, 126.4, 126.3$_9$, 126.3$_4$, 126.3$_1$, 126.1, 114.8, 114.7, 71.7, 61.8, 60.8, 55.5, 17.3, 16.0, 10.8; FAB-MS (matrix: *m*-NBA) *m/z* 554 ([M-Br$^-$]$^+$ 50%); Anal. Calcd. for $C_{39}H_{40}BrNO_2 \cdot 0.35CHCl_3$: C, 69.87; H, 6.01; N, 2.07. Found: C, 69.94; H, 6.28; N, 2.28.

(*R*)-2,10-Bis(4-methoxyphenyl)-6-(1-phenylethyl)-6-propyl-6,7-dihydro-5*H*-dibenzo[*c,e*]azepin-6-ium bromide ((*R*)-2f)

Following general procedure was carried out with **1** (70.0 mg, 0.127 mmol), (*R*)-*N*-(1-phenylethyl)propan-1-amine [1] (23.1 mg, 0.142 mmol) and K_2CO_3 (110 mg, 0.799 mmol) in CH_3CN (4 mL) at 85 °C for 23 h. Purification using column chromatography ($CHCl_3$/MeOH, v/v 5:1) afforded ammonium salt (*R*)-**2f** (60.4 mg, 74% yield) as pale yellow solid: m.p. 124.3–125.0 °C; ^{1}H NMR (400 MHz, $CDCl_3$) δ 8.01 (d, J = 1.8 Hz, 1H), 7.97 (d, J = 1.8 Hz, 1H), 7.82–7.70 (m, 7H), 7.61 (dd, J_1 = 7.9 Hz, J_2 = 1.8 Hz, 1H), 7.95–7.34 (m, 4H), 7.08–7.02 (m, 4H), 5.12 (q, J = 7.0 Hz, 1H), 4.61 (dd, J_1 = 13.0 Hz, J_2 =13.0 Hz, 2H), 4.16 (dd, J_1 = 13.0 Hz, J_2 = 13.0 Hz, 2H), 3.82 (s, 6H), 3.59–3.49 (m, 1H), 3.27–3.14 (m, 1H), 2.04–1.90 (m, 5H), 0.90 (t, J = 7.3 Hz); ^{13}C NMR (100 MHz, $CDCl_3$) δ 159.8$_3$, 159.8$_0$, 142.4, 142.1, 141.0$_6$, 140.9, 134.2, 133.2, 132.8, 131.5, 131.0, 130.2, 129.0, 128.5, 128.3, 128.2$_4$, 128.2$_1$, 127.1, 126.4, 126.3$_5$, 126.3$_1$, 126.1, 114.8, 114.7,

71.7, 61.7, 60.8, 55.5, 17.3, 16.0, 10.8; FAB-MS (matrix: *m*-NBA) *m/z* 554 ($[M\text{-}Br^-]^+$ 40%); Anal. Calcd. for $C_{39}H_{40}BrNO_2 \cdot 0.35CHCl_3$: C, 69.87; H, 6.01; N, 2.07. Found: C, 69.65; H, 6.31; N, 2.13.

(*S*)-2,10-Bis(4-methoxyphenyl)-6-methyl-6-(1-phenylethyl)-6,7-dihydro-5*H*-dibenzo[*c,e*]azepin-6-ium bromide ((*S*)-2g)

Following general procedure was carried out with **1** (70.0 mg, 0.127 mmol), (*S*)-*N*-methyl-1-phenylethylamine (20.0 μL, 0.136 mmol) and K_2CO_3 (113.0 mg, 0.816 mmol) in CH_3CN (4 mL) at 65 °C for 23 h. Purification using column chromatography ($CHCl_3$/MeOH, v/v 20:1) afforded ammonium salt (*S*)-**2g** (56.5 mg, 74% yield) as pale yellow solid: m.p. 149.0–150.0 °C; ^{1}H NMR (400 MHz, $CDCl_3$) δ 8.08–8.07 (m, 2H), 7.83–7.69 (m, 10H), 7.55–7.51 (m, 3H), 7.09–7.04 (m, 4H), 4.92 (q, J = 6.8 Hz, 1H), 4.58 (d, J = 13.0 Hz, 1H), 4.14 (d, J = 13.0 Hz, 2H), 3.83 (s, 6H), 3.69 (d, J = 13.0 Hz, 1H), 3.12 (s, 3H),1.97, (d, J = 6.8Hz, 3H); ^{13}C NMR (100 MHz, $CDCl_3$) δ 159.9, 142.7$_3$, 142.7$_1$, 141.3$_4$, 141.3$_3$, 133.6, 132.8, 132.7, 131.6, 131.5, 131.0, 130.3, 129.1, 128.34, 128.32, 126.5, 126.4, 126.3, 126.2, 114.8, 71.3, 62.6, 61.2, 55.5, 44.3, 16.1; FAB-MS (matrix: *m*-NBA) *m/z* 526 ($[M\text{-}Br^-]^+$ 90%); Anal. Calcd. for $C_{37}H_{36}BrNO_2 \cdot 0.25CHCl_3$: C, 70.30; H, 5.74; N, 2.20. Found: C, 70.10; H, 6.04; N, 2.21.

(*R*)-2'-(Hydroxymethyl)-2,10-bis(4-methoxyphenyl)-5,7-dihydrospiro[dibenzo[*c,e*]azepine-6,1'-pyrrolidin]-6-ium bromide ((*R*)-2h)

Following general procedure was carried out with **1** (70.0 mg, 0.127 mmol), D-prolinol (13.0 μL, 0.134 mmol) and K_2CO_3 (112.0 mg, 0.810 mmol) in CH_3CN (4 mL) at 65 °C for 23 h. Purification using column chromatography ($CHCl_3$/MeOH, v/v 20:1) afforded ammonium salt (*R*)-**2h** (52.6 mg, 73% yield) as colorless solid: m.p. 302.1 °C (dec.); ^{1}H NMR (400 MHz, $CDCl_3$) δ 8.05–8.04 (m, 2H), 7.83–7.56 (m, 8H), 7.60 (dt, J_1 = 8.6 Hz, J_2 = 3.0, 4H), 5.18 (t, J = 4.6 Hz, 1H), 4.48 (dd, J_1 = 13.0 Hz, J_2 =13.0 Hz, 2H), 4.22–4.06 (m, 3H), 3.95–3.65 (m, 10H), 2.59–2.50 (m, 1H), 2.32–2.13 (m, 3H); ^{13}C NMR (100 MHz, $CDCl_3$) δ 159.8$_1$, 159.7$_9$, 142.7, 142.4, 141.2, 132.3, 132.1, 131.6$_4$, 131.6$_2$, 128.3$_2$, 128.2$_9$, 127.3, 127.0, 126.5, 126.4, 126.3, 114.8, 75.2, 63.4, 62.9, 59.3, 57.1, 55.5, 24.9, 19.8; FAB-MS (matrix: *m*-NBA) *m/z* 492 ($[M\text{-}Br^-]^+$ 100%); Anal. Calcd. for $C_{33}H_{34}BrNO_3 \cdot 0.18CH_2Cl_2$: C, 65.78; H, 5.76; N, 2.29. Found: C, 65.82; H, 5.59; N, 2.45.

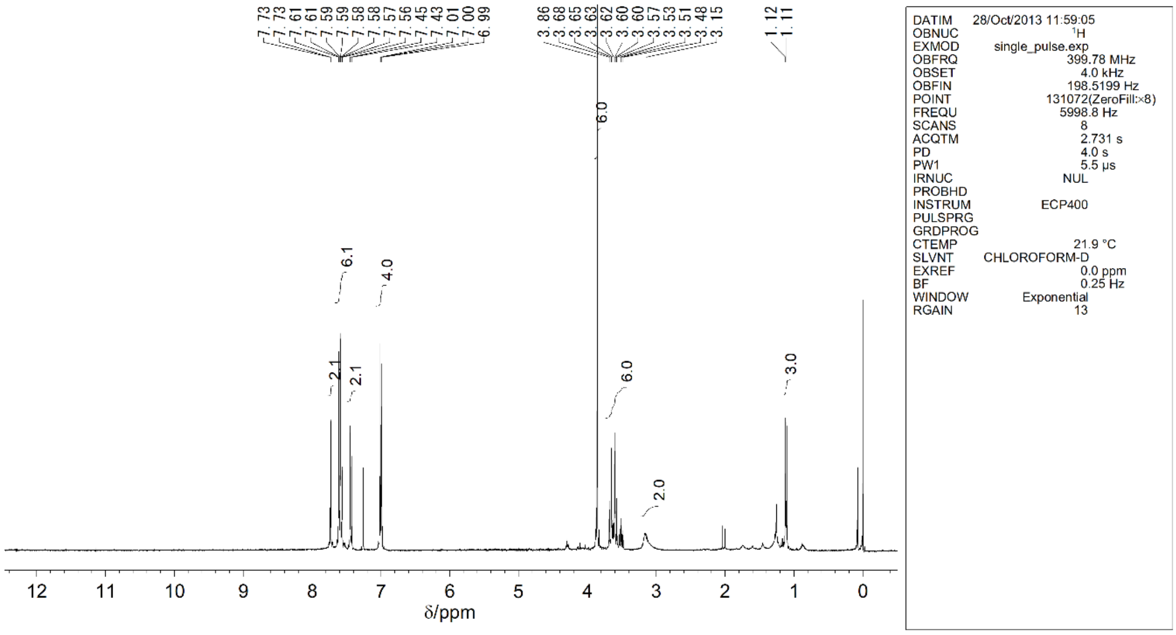

Figure S1. ^{1}H NMR Spectrum (400 MHz, $CDCl_3$) of (*S*)-**2a**

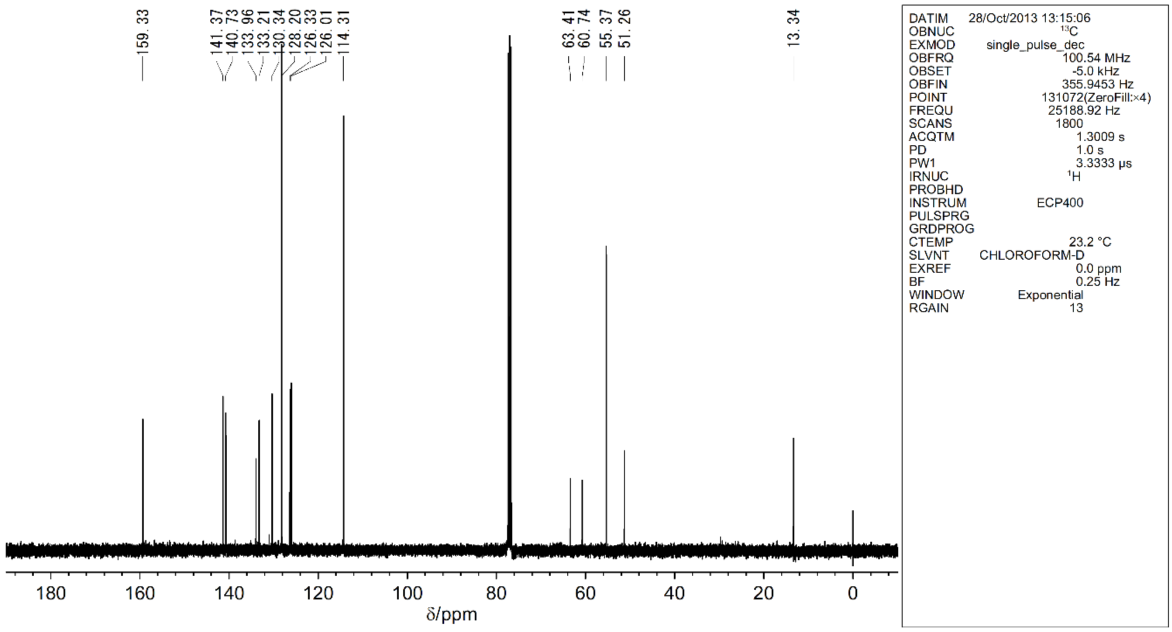

Figure S2. ^{13}C NMR Spectrum (100 MHz, $CDCl_3$) of (*S*)-**2a**

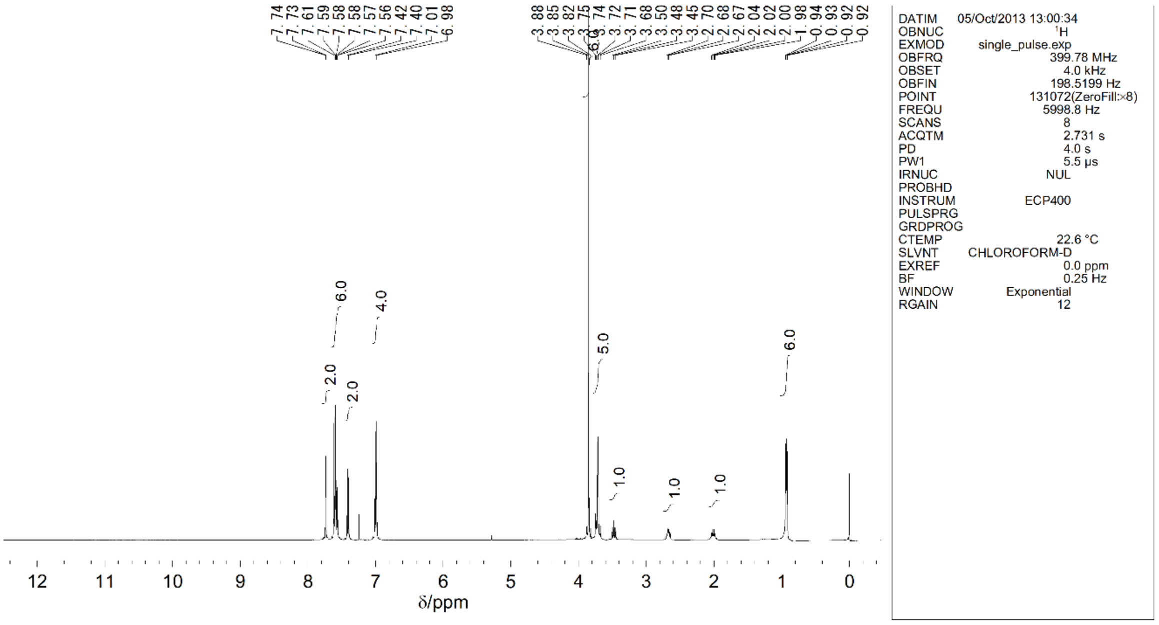

Figure S3. ^{1}H NMR Spectrum (400 MHz, $CDCl_3$) of (*S*)-**2b**

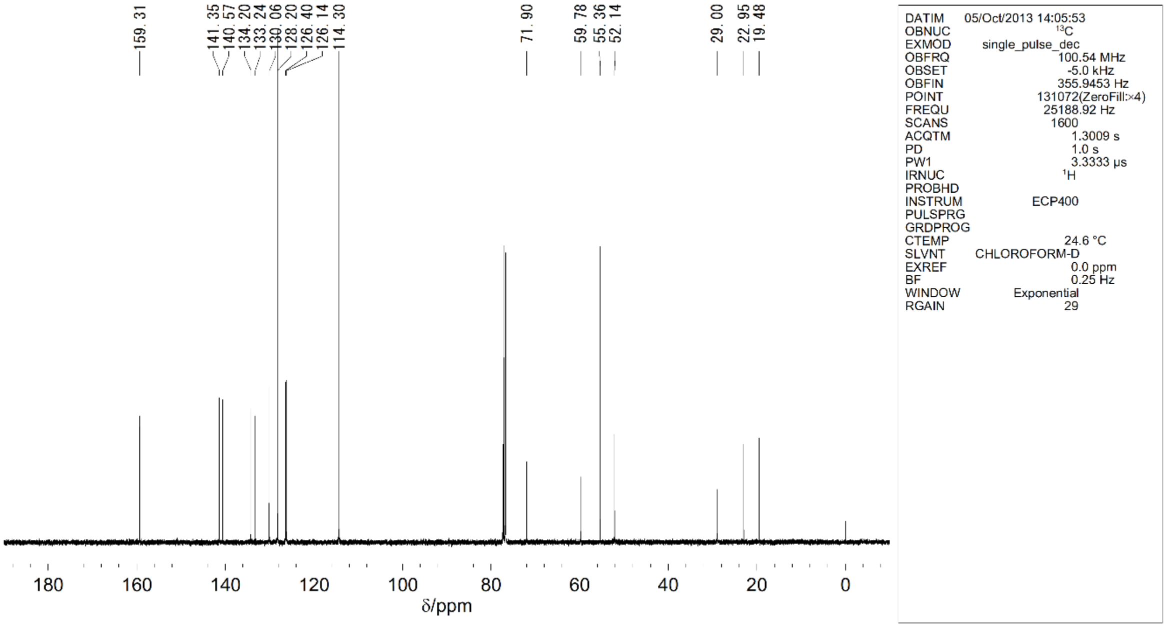

Figure S4. ^{13}C NMR Spectrum (100 MHz, $CDCl_3$) of (*S*)-**2b**

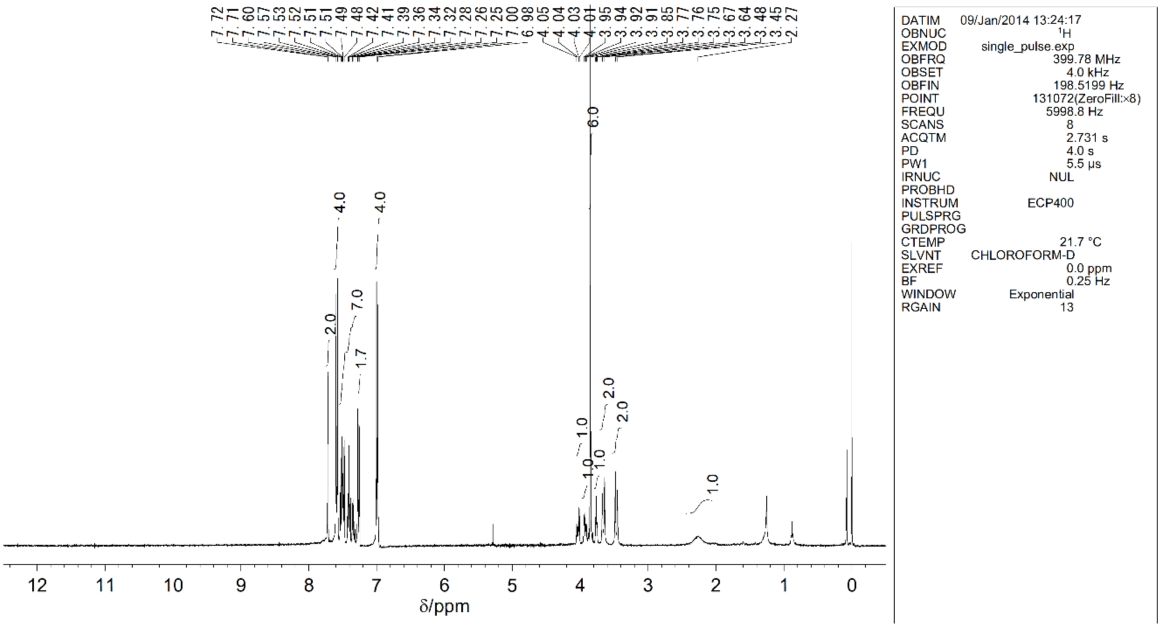

Figure S5. ^{1}H NMR Spectrum (400 MHz, $CDCl_3$) of (*S*)-**2c**

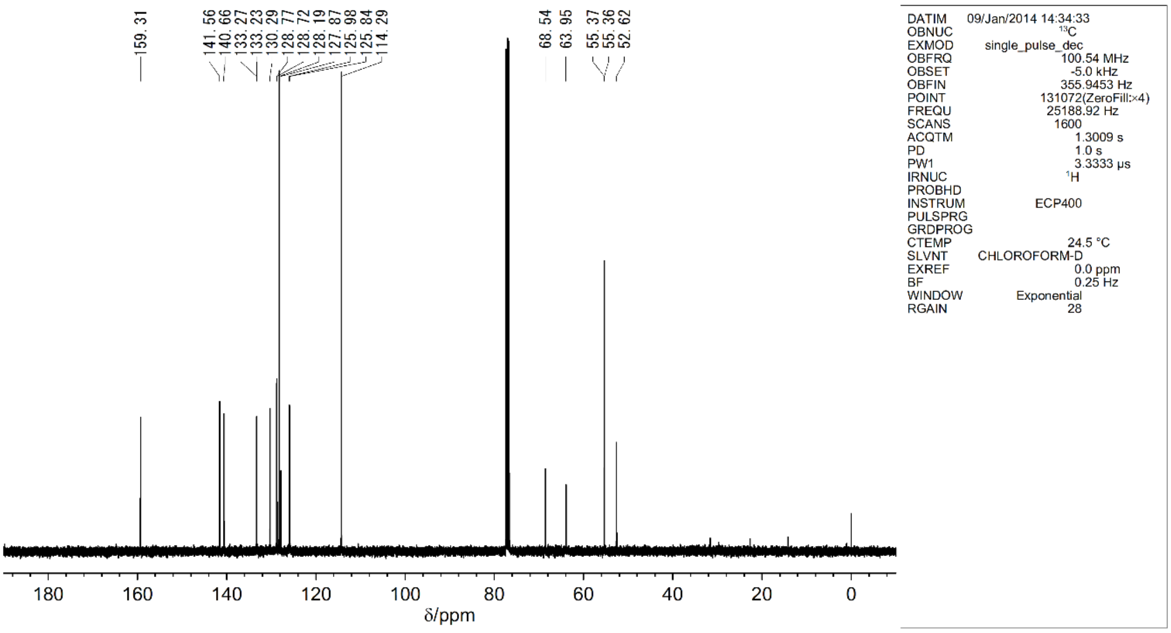

Figure S6. ^{13}C NMR Spectrum (100 MHz, $CDCl_3$) of (*S*)-**2c**

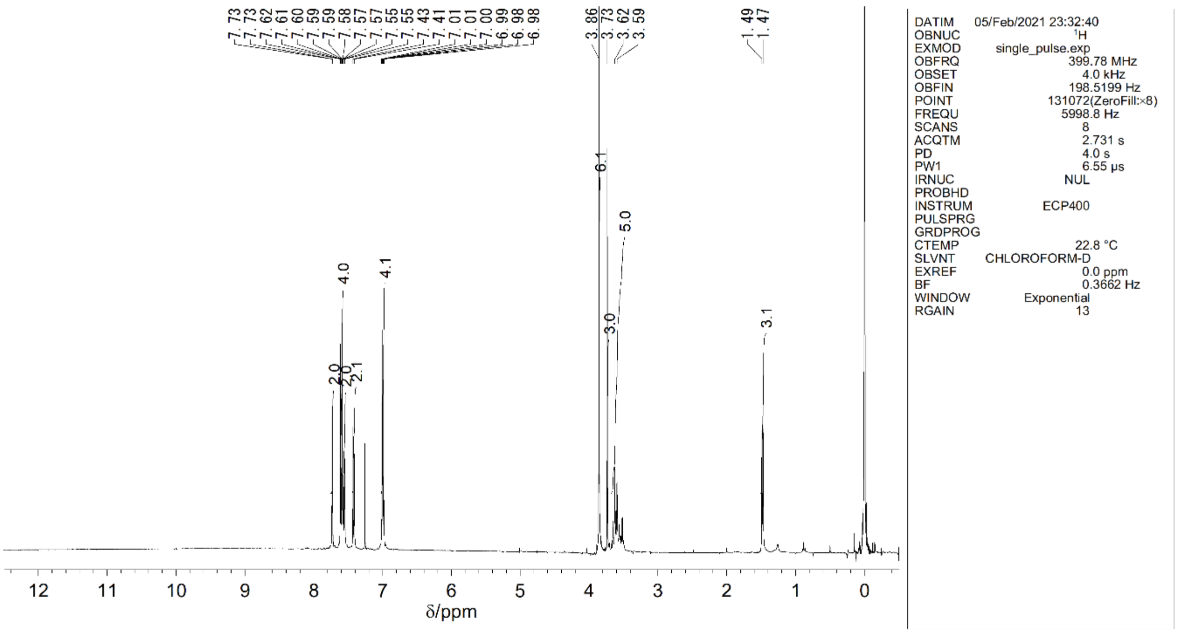

Figure S7. ^{1}H NMR Spectrum (400 MHz, $CDCl_3$) of (*S*)-**2d**

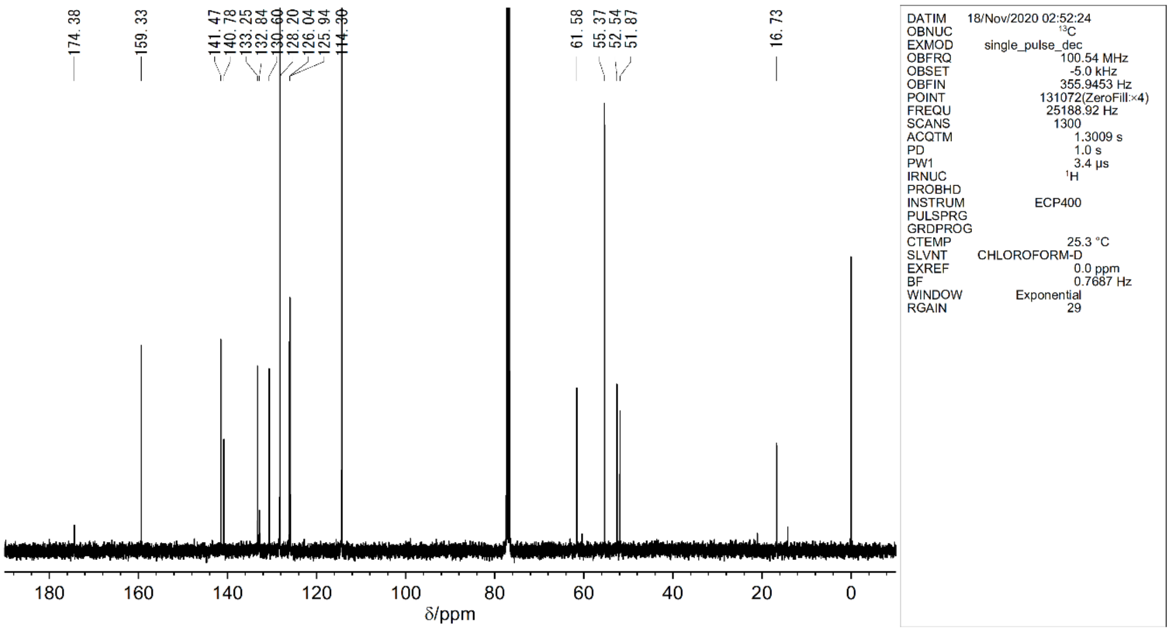

Figure S8. ^{13}C NMR Spectrum (100 MHz, $CDCl_3$) of (*S*)-**2d**

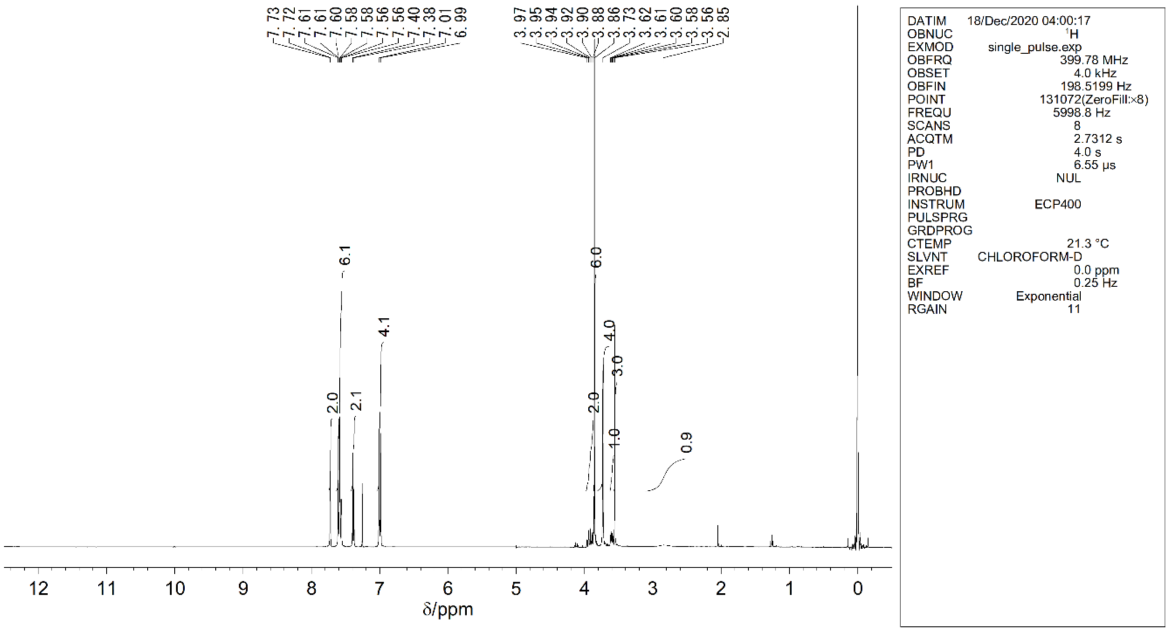

Figure S9. ^{1}H NMR Spectrum (400 MHz, $CDCl_3$) of (*S*)-**2e**

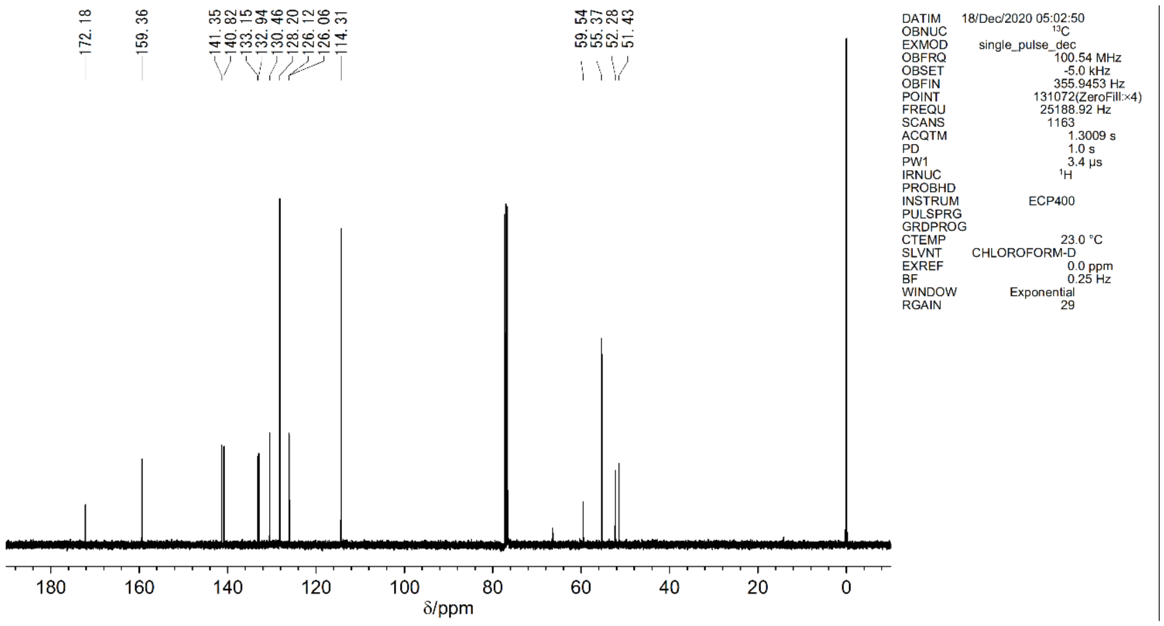

Figure S10. ^{13}C NMR Spectrum (100 MHz, $CDCl_3$) of (*S*)-**2e**

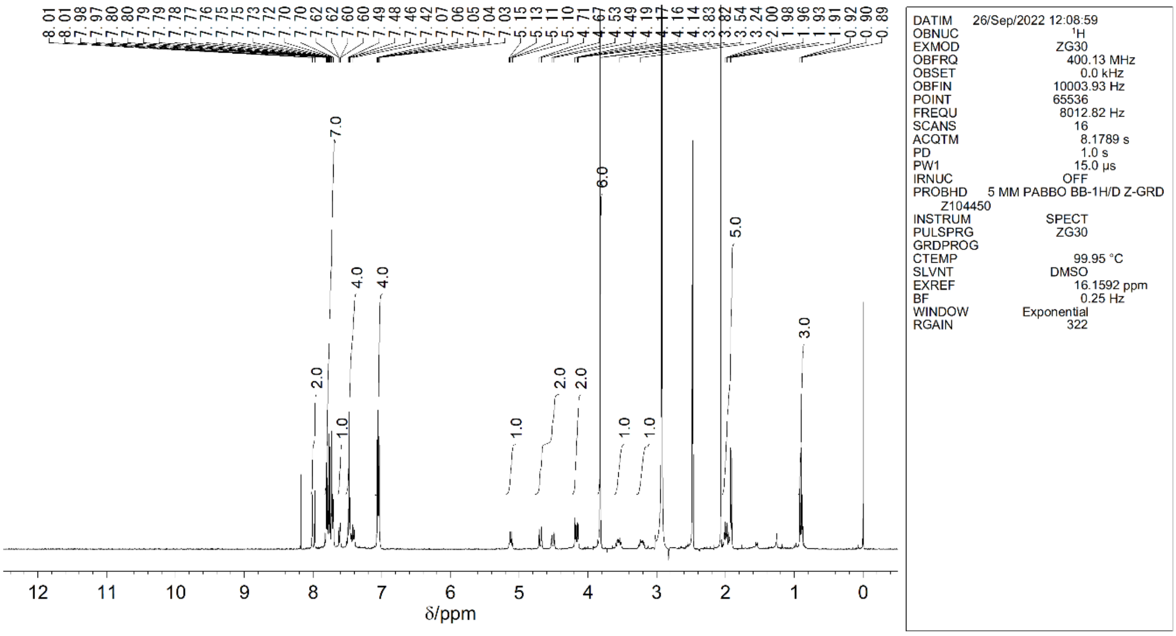

Figure S11. ^{1}H NMR Spectrum (400 MHz, DMSO-d_6, 373K) of (*S*)-**2f**

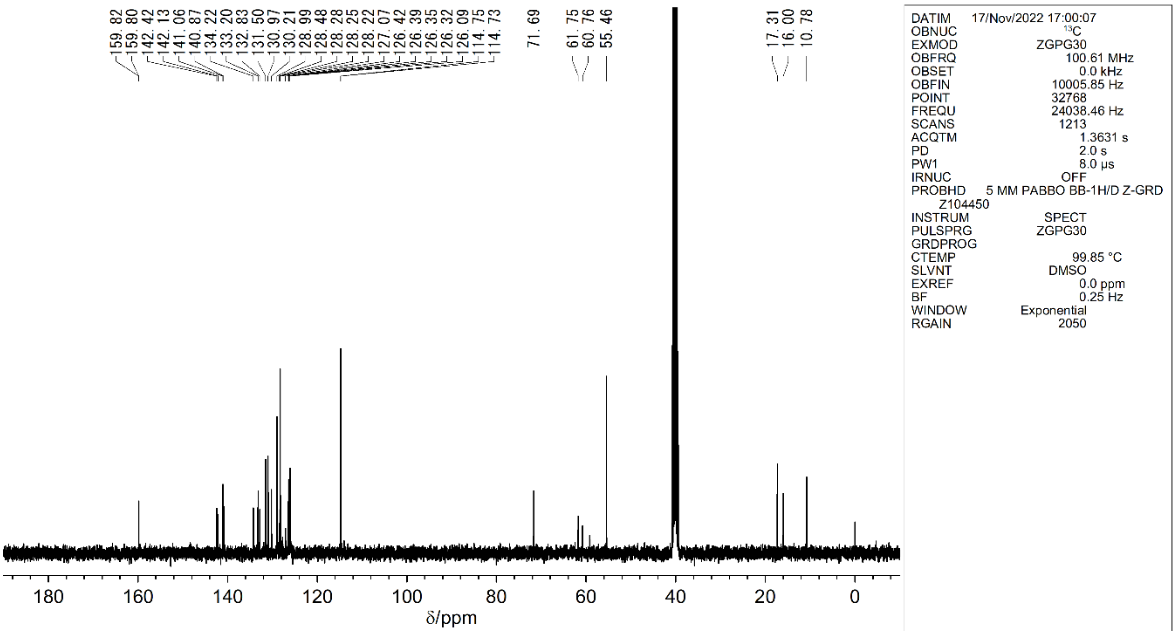

Figure S12. ^{13}C NMR Spectrum (100 MHz, DMSO-d_6, 373K) of (*S*)-**2f**

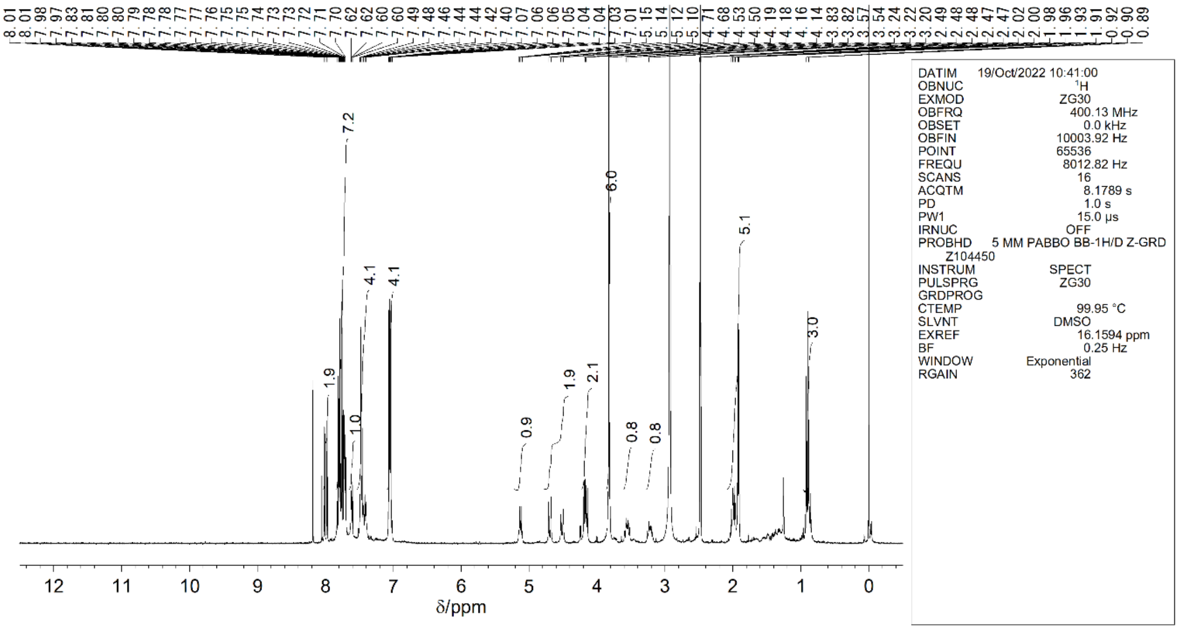

Figure S13. ^{1}H NMR Spectrum (400 MHz, DMSO-d_6, 373K) of (*R*)-**2f**

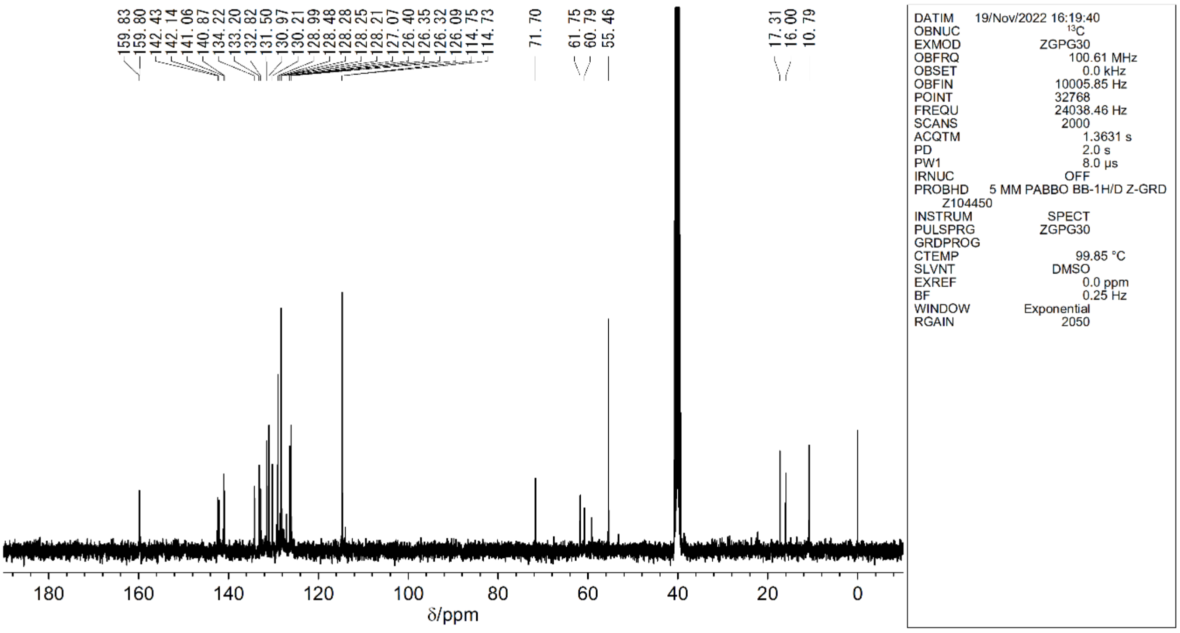

Figure S14. ^{13}C NMR Spectrum (100 MHz, DMSO-d_6, 373K) of (*R*)-**2f**

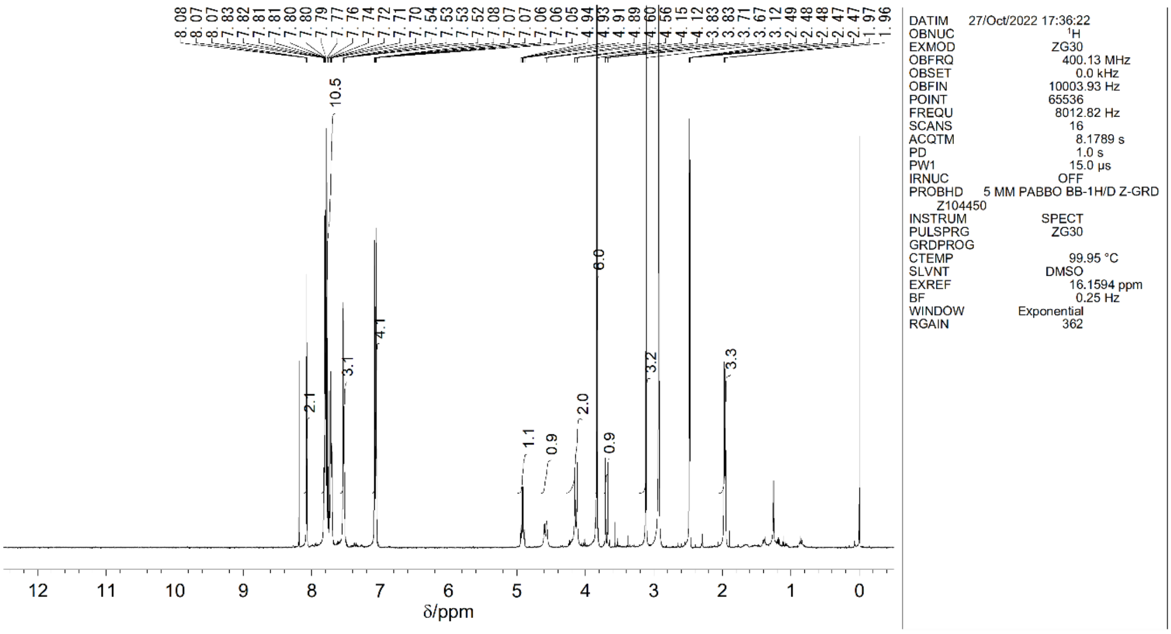

Figure S15. ^{1}H NMR Spectrum (400 MHz, DMSO-d_6, 373K) of (*S*)-**2g**

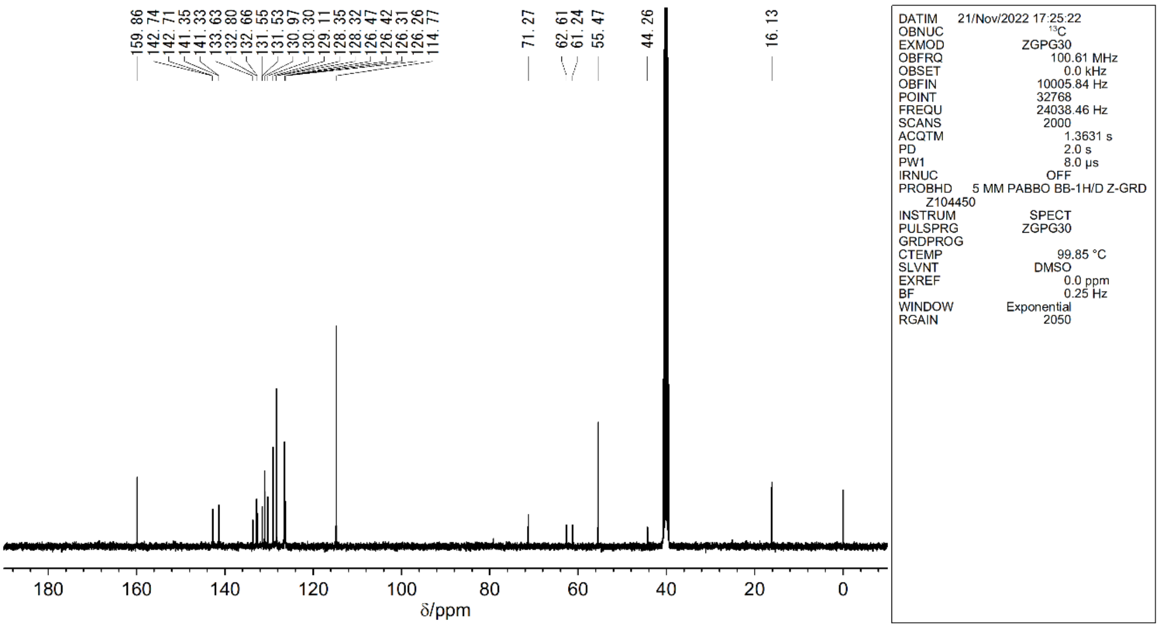

Figure S16. ^{13}C NMR Spectrum (100 MHz, DMSO-d_6, 373K) of (*S*)-**2g**

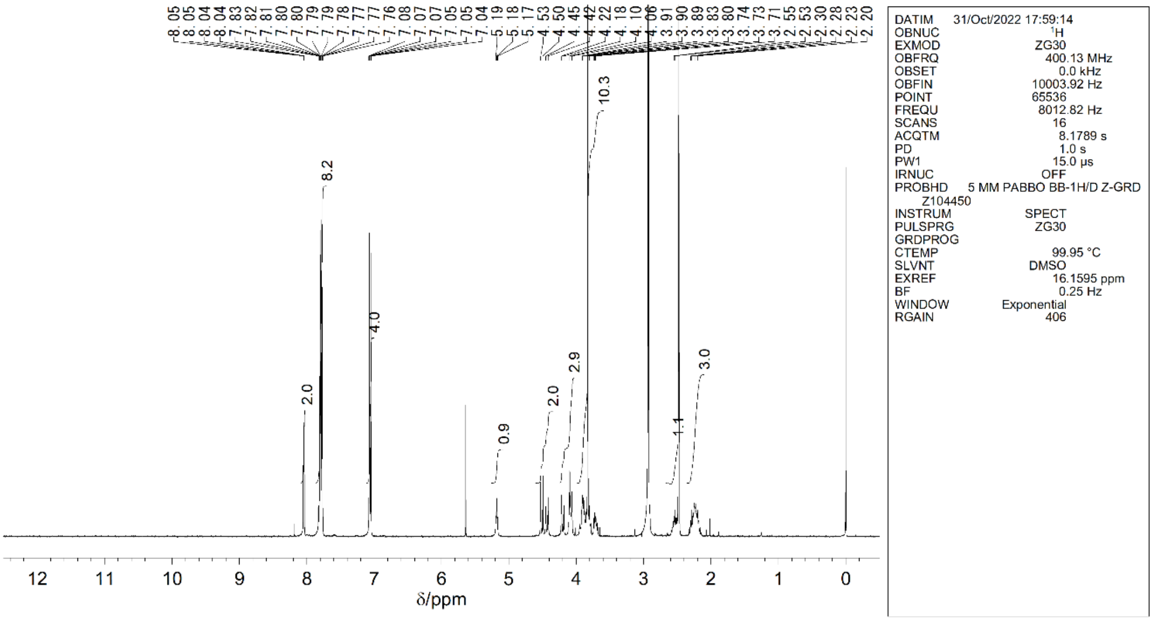

Figure S17. ^{1}H NMR Spectrum (400 MHz, DMSO-d_6, 373K) of (*R*)-**2h**

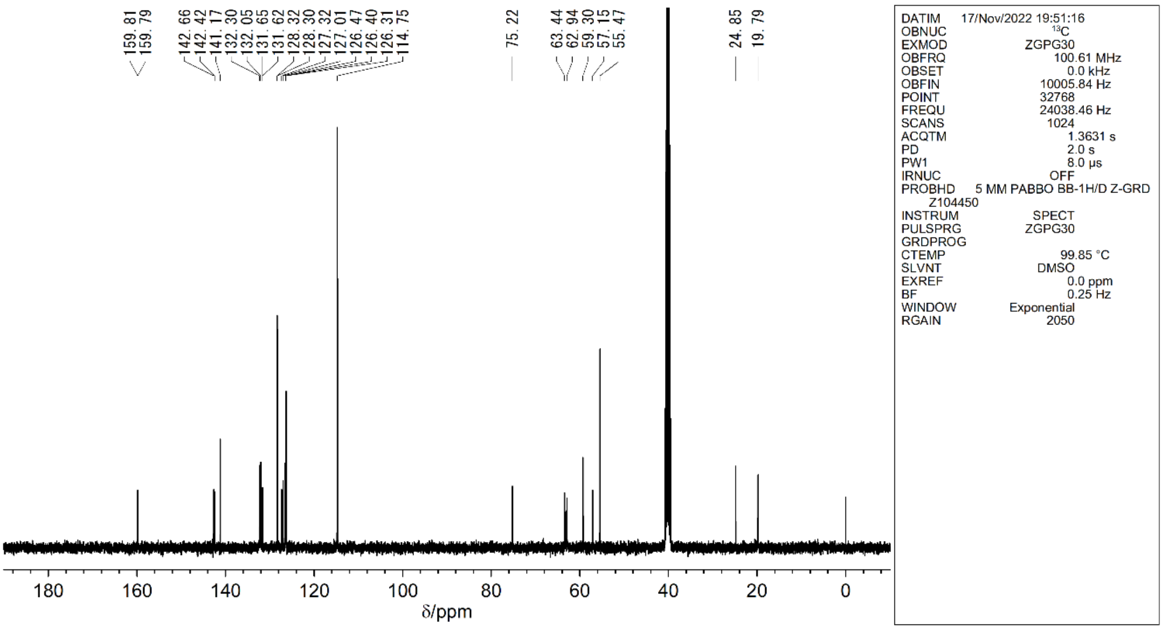

Figure S18. ^{13}C NMR Spectrum (100 MHz, DMSO-d_6, 373K) of (*R*)-**2h**

Table S1. CD spectral data of (*S*)-**2a–e**,**i–n**[a]

Entry	Predicted helicity	$\Delta\varepsilon_1$[b] (λ [nm])	$\Delta\varepsilon_2$[b] (λ [nm])	Experimental helicity	CD amplitude (A_{CD} value)[c]
1	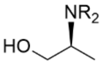 (*S*)-**2a** 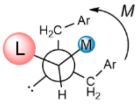	–0.6 (277.6)	+1.7 (257.6)	*M*	–2.3
2	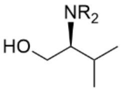 (*S*)-**2b** 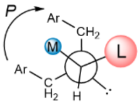	+3.1 (280.0)	–0.9 (255.6)	*P*	+4.0
3	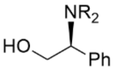 (*S*)-**2c** 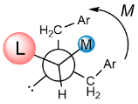	–8.6 (287.2)	+10.6 (261.2)	*M*	–19.2
4	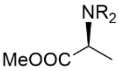 (*S*)-**2d** 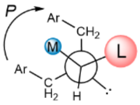	+5.9 (285.6)	–4.4 (261.1)	*P*	+10.3
5	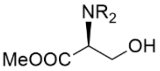 (*S*)-**2e** 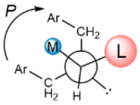	+8.2 (283.6)	–5.8 (257.8)	*P*	+14.0
6[c]	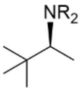 (*S*)-**2i** 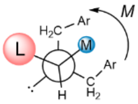	–9.2 (278.8)	+8.5 (256.2)	*M*	–17.7
7[c]	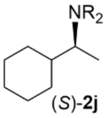 (*S*)-**2j** 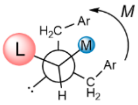	–3.5 (279.4)	+4.0 (257.2)	*M*	–7.5
8[c]	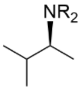 (*S*)-**2k** 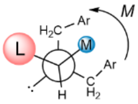	–2.4 (281.4)	+3.5 (255.0)	*M*	–5.9
9[c]	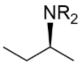 (*S*)-**2l** 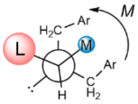	–0.6 (282.6)	+1.2 (260.2)	*M*	–1.8
10[c]	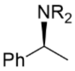 (*S*)-**2m** 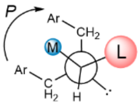	+9.8 (286.8)	–10.6 (261.2)	*P*	+20.4
11[c]	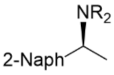 (*S*)-**2n** 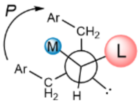	+7.2 (287.4)	–15.0 (258.4)	*P*	+22.2

[a] All CD data were measured in CH_3CN, 2 x 10^{-4} M concentration using 1 mm CD cell at 293 K. [b] $\Delta\varepsilon_1$ and $\Delta\varepsilon_2$ are intensities of first and second Cotton effects. [c] A_{CD} value: $A_{CD} = \Delta\varepsilon_1 - \Delta\varepsilon_2$, where $\Delta\varepsilon_1$ and $\Delta\varepsilon_2$ are intensities of first and second Cotton effects, respectively. [c] Kuwahara, S.; Nakamura, M.; Yamaguchi, A.; Ikeda, M.; Habata, Y. *Org. Lett.* **2013**, 15, 5738–5741.

Table S2. CD spectral data of (*S*)-**2f–h**[a].

Entry	Compound	$\Delta\varepsilon_1$[b] (λ [nm])	$\Delta\varepsilon_2$[b] (λ [nm])	Experimental helicity	CD amplitude (A_{CD} value)[c]
1	(*S*)-**2f**	–7.0 (292.6)	+5.5 (266.0)	*M*	–12.5
2	(*S*)-**2g**	–15.3 (292.3)	+6.3 (265.1)	*M*	–21.6
3	(*S*)-**2h**	+19.3 (291.1)	–13.5 (265.6)	*P*	+32.8

[a] All CD data were measured in CH_3CN, 2 x 10^{-4} M concentration using 1 mm CD cell at 293 K. [b] $\Delta\varepsilon_1$ and $\Delta\varepsilon_2$ are intensities of first and second Cotton effects. [c] A_{CD} value: $A_{CD} = \Delta\varepsilon_1 - \Delta\varepsilon_2$, where $\Delta\varepsilon_1$ and $\Delta\varepsilon_2$ are intensities of first and second Cotton effects, respectively.

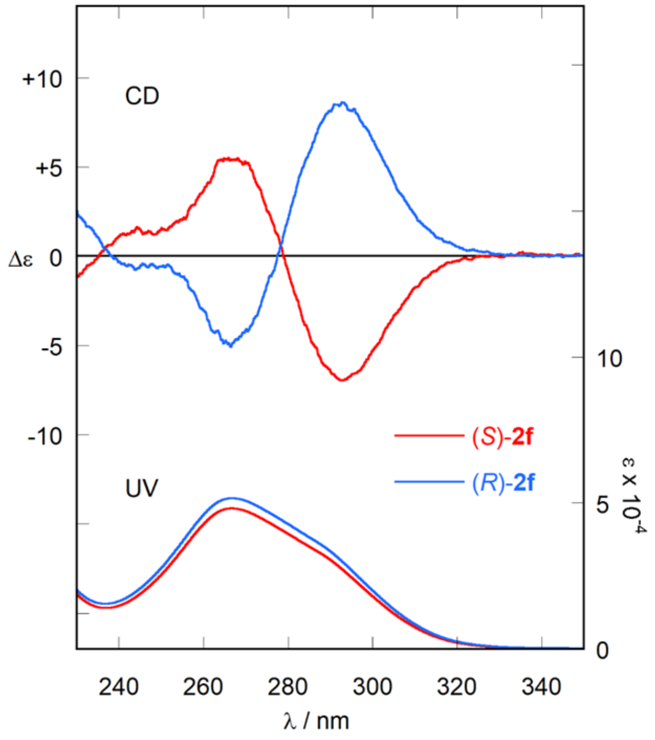

Figure S19. CD and UV spectra of (*S*)-**2f** and (*R*)-**2f** (2 × 10^{-4} M in CH_3CN, 293 K).

Theoretical calculations at B3LYP/6-31G* level

Table S3. Calculated conformers of (*S*)-**3a** at B3LYP/6-31G* level.

Entry	Conformer	Helicity	Dihedral angle[a]	*E*, kJ/mol	*K*[b]	Population, %
1	#M1	*M*	−42.64	0.00	1.00	47.9
2	#P1	*P*	43.25	0.25	0.90	43.2
3	#P3	*P*	43.01	5.52	0.10	5.0
4	#M3	*M*	−42.75	6.14	0.08	3.9

[a] Dihedral angle of C6-C1-C1'-C6'. [b] Equilibrium constant at 298 K.

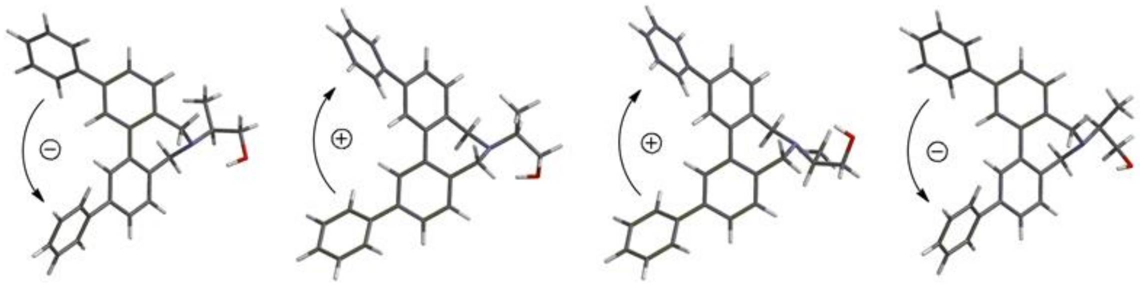

Figure S20. Four major conformers of (*S*)-**3a** at B3LYP/6-31G* level.

Table S4. Calculated conformers of (*S*)-**3b** at B3LYP/6-31G* level.

Entry	Conformer	Helicity	Dihedral angle[a]	*E*, kJ/mol	*K*[b]	Population, %
1	#M1	*M*	−41.67	0.00	1.00	23.1
2	#P4	*P*	42.51	1.05	0.65	15.0
3	#P6	*P*	42.71	2.76	0.32	7.4
4	#P92	*P*	43.56	3.09	0.28	6.5
5	#P73	*P*	43.17	3.18	0.27	6.3
6	#M3	*M*	−43.71	3.30	0.26	6.0
7	#P1	*P*	41.34	3.43	0.24	5.7
8	#P39	*P*	42.54	3.43	0.24	5.7
9	#P2	*P*	42.94	3.51	0.24	5.5
10	#M5	*M*	−41.63	4.01	0.19	4.4
11	#P12	*P*	42.19	4.56	0.15	3.6
12	#P22	*P*	42.17	5.69	0.10	2.2
13	#P25	*P*	42.45	5.69	0.10	2.2
14	#M13	*M*	−42.23	6.23	0.08	1.8
15	#M26	*M*	−43.51	6.40	0.07	1.7
16	#P99	*P*	43.45	7.61	0.04	1.0
17	#P8	*P*	43.17	8.45	0.03	0.7
18	#M9	*M*	−43.15	9.87	0.02	0.4

[a] Dihedral angle of C6-C1-C1'-C6'. [b] Equilibrium constant at 298 K.

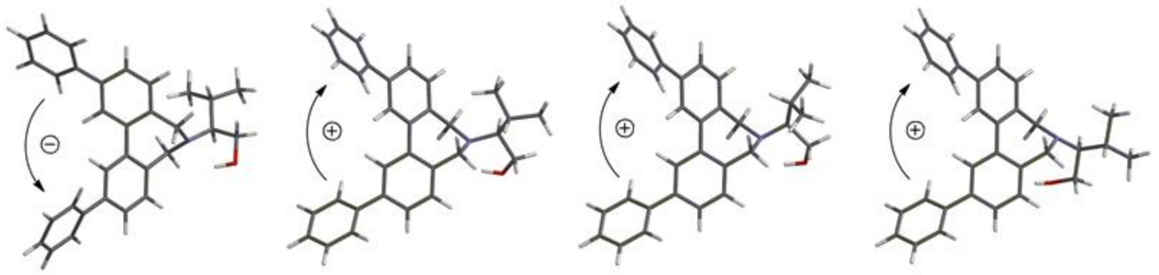

#M1 (23.1%) #P4 (15.0%) #P6 (7.4%) #P92 (6.5%)

Figure S21. Four major conformers of (*S*)-**3b** at B3LYP/6-31G* level.

Table S5. Calculated conformers of (*S*)-**3c** at B3LYP/6-31G* level.

Entry	Conformer	Helicity	Dihedral angle[a]	*E*, kJ/mol	*K*[b]	Population, %
1	#M3	*M*	−45.52	0.00	1.00	48.7
2	#P1	*P*	43.01	2.64	0.34	16.5
3	#M6	*M*	−42.96	3.80	0.21	10.2
4	#P6	*P*	42.01	4.69	0.15	7.1
5	#M10	*M*	−42.39	5.63	0.10	4.8
6	#M16	*M*	−42.06	5.72	0.10	4.7
7	#M7	*M*	−41.95	5.81	0.09	4.5
8	#P20	*P*	43.33	7.50	0.05	2.2
9	#P13	*P*	42.97	8.83	0.03	1.3

[a] Dihedral angle of C6-C1-C1'-C6'. [b] Equilibrium constant at 298 K.

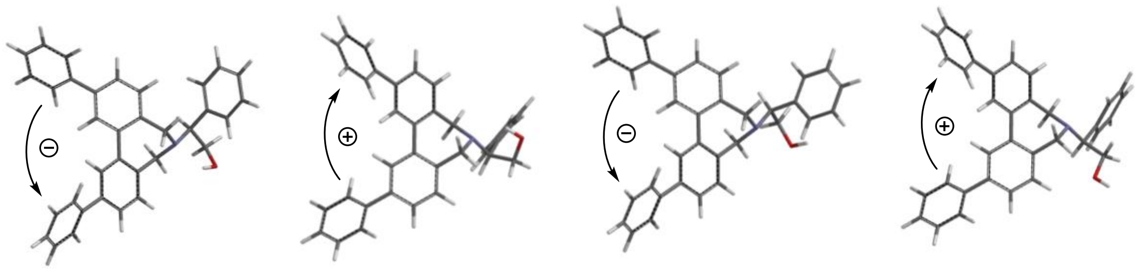

#M3 (48.7%) #P1 (16.5%) #M6 (10.2%) #P6 (7.1%)

Figure S22. Three major conformers of (*S*)-**3c** at B3LYP/6-31G* level.

Table S6. Calculated conformers of (*S*)-**3d** at B3LYP/6-31G* level.

Entry	Conformer	Helicity	Dihedral angle[a]	*E*, kJ/mol	*K*[b]	Population, %
1	#P1	*P*	42.31	0.00	1.00	63.2
2	#M1	*M*	−42.62	3.15	0.27	17.4
3	#M3	*M*	−42.65	4.69	0.15	9.2
4	#P5	*P*	42.02	5.93	0.09	5.5
5	#M9	*M*	−42.61	8.18	0.03	2.2
6	#P3	*P*	43.06	9.38	0.02	1.3
7	#P9	*P*	43.82	9.92	0.02	1.1

[a] Dihedral angle of C6-C1-C1'-C6'. [b] Equilibrium constant at 298 K.

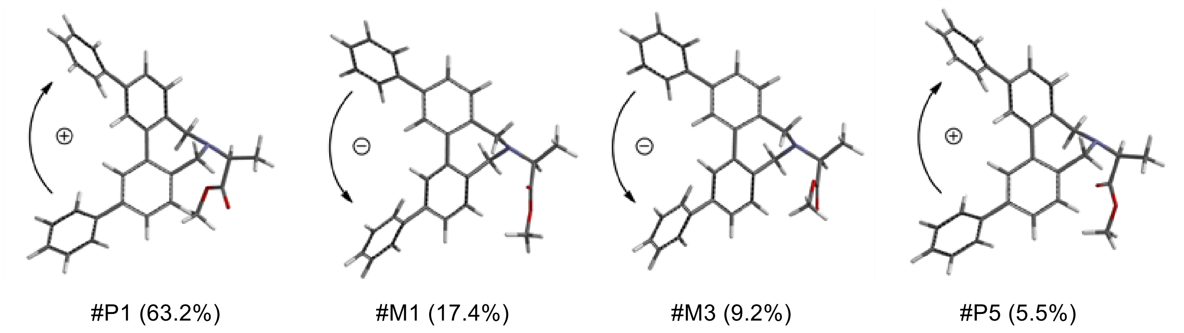

Figure S23. Four major conformers of (*S*)-**3d** at B3LYP/6-31G* level.

Table S7. Calculated conformers of (*S*)-**3e** at B3LYP/6-31G* level.

Entry	Conformer	Helicity	Dihedral angle[a]	*E*, kJ/mol	*K*[b]	Population, %
1	#P12	*P*	42.77	0	1.00	34.7
2	#P2	*P*	42.08	0.27	0.90	31.0
3	#P14	*P*	41.69	2.48	0.36	12.5
4	#M19	*M*	−43.55	4.15	0.18	6.3
5	#M1	*M*	−43.05	4.32	0.17	5.9
6	#M20	*M*	−42.27	4.59	0.15	5.3
7	#M3	*M*	−43.51	7.63	0.04	1.5
8	#P4	*P*	42.00	8.12	0.04	1.2
9	#M56	*M*	−43.15	8.76	0.03	1.0
10	#M15	*M*	−42.54	9.71	0.02	0.6

[a] Dihedral angle of C6-C1-C1'-C6'. [b] Equilibrium constant at 298 K.

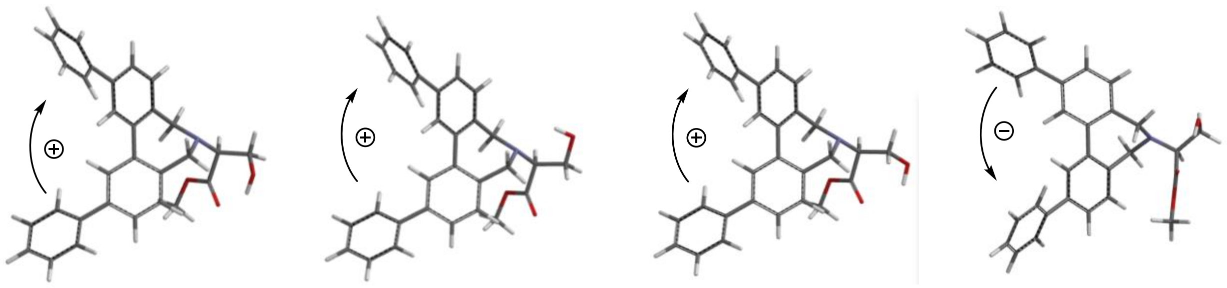

#P12 (34.7%) #P2 (31.0%) #P14 (12.5%) #M19 (6.3%)

Figure S24. Four major conformers of (*S*)-**3e** at B3LYP/6-31G* level.

Table S8. Calculated conformers of (*S*)-**3f** at B3LYP/6-31G* level.

Entry	Conformer	Helicity	Dihedral angle[a]	*E*, kJ/mol	*K*[b]	Population, %
1	#M3	*M*	−41.74	0.00	1.00	53.5
2	#P1	*P*	40.44	2.95	0.30	15.9
3	#M2	*M*	−41.81	3.57	0.23	12.4
4	#P4	*P*	42.39	5.02	0.13	6.8
5	#P5	*P*	42.08	5.95	0.09	4.7
6	#M6	*M*	−43.02	7.42	0.05	2.5
7	#M1	*M*	−40.24	8.54	0.03	1.6
8	#P2	*P*	39.83	8.95	0.03	1.4
9	#M5	*M*	−40.90	9.18	0.02	1.2

[a] Dihedral angle of C6-C1-C1'-C6'. [b] Equilibrium constant at 298 K.

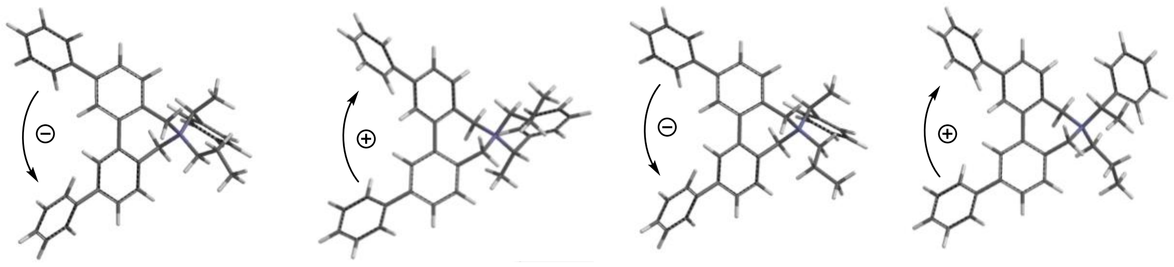

#M3 (53.5%) #P1 (15.9%) #M2 (12.4%) #P4 (6.8%)

Figure S25. Four major conformers of (*S*)-**3f** at B3LYP/6-31G* level.

Table S9. Calculated conformers of (*S*)-**3g** at B3LYP/6-31G* level.

Entry	Conformer	Helicity	Dihedral angle[a]	*E*, kJ/mol	*K*[b]	Population, %
1	#M2	*M*	−43.39	0.00	1.00	73.5
2	#P1	*P*	42.25	2.49	0.36	26.5

[a] Dihedral angle of C6-C1-C1’-C6’. [b] Equilibrium constant at 298 K.

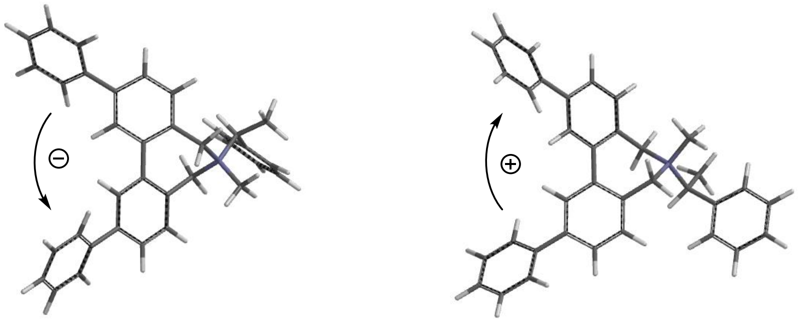

#M2 (73.5%) #P1 (26.5%)

Figure S26. Two major conformers of (*S*)-**3g** at B3LYP/6-31G* level.

Table S10. Calculated conformers of (*R*)-**3h** at B3LYP/6-31G* level.

Entry	Conformer	Helicity	Dihedral angle[a]	*E*, kJ/mol	*K*[b]	Population, %
1	#P3	*P*	42.49	0.00	1.00	57.3
2	#M4	*M*	−42.59	4.63	0.15	8.6
3	#M7	*M*	−41.95	5.10	0.12	7.1
4	#P1	*P*	42.55	5.57	0.10	5.8
5	#P2	*P*	43.32	5.93	0.09	5.0
6	#P11	*P*	43.92	6.27	0.08	4.4
7	#P6	*P*	43.76	6.69	0.06	3.7
8	#P22	*P*	43.92	6.83	0.06	3.5
9	#P5	*P*	43.10	7.99	0.04	2.2
10	#P8	*P*	39.96	8.71	0.03	1.6
11	#P9	*P*	40.57	9.90	0.02	1.0

[a] Dihedral angle of C6-C1-C1’-C6’. [b] Equilibrium constant at 298 K.

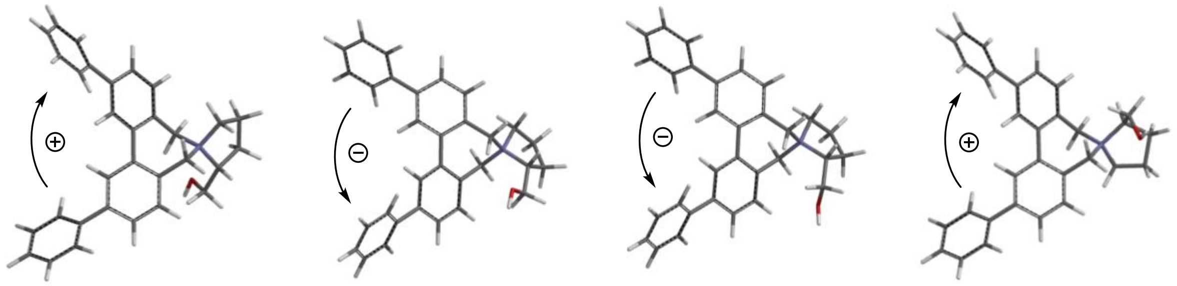

#P3 (57.3%) #M4 (8.6%) #M7 (7.1%) #P1 (5.8%)

Figure S27. Four major conformers of (*R*)-**3h** at B3LYP/6-31G* level.

Table S11. Comparison of the excess of *P* conformer and observed CD amplitude at B3LYP/6-31G* level.

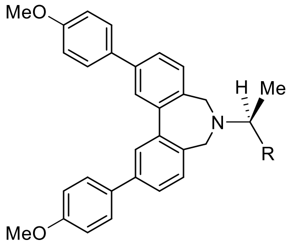

(*S*)-**2i**: R = *t*-Bu
(*S*)-**2j**: R = cyclohexyl
(*S*)-**2k**: R = *i*-Pr
(*S*)-**2l**: R = Et
(*S*)-**2m**: R = Ph
(*S*)-**2n**: R = 2-Naph

Entry	Compound	Calcurated ratio (*P/M*)	Excess of *P* confomer, %[a]	Observed CD amplitude (A_{CD} value)[b]
1	(*S*)-**2a**	48.2:51.8	–3.6	–2.3
2	(*S*)-**2b**	62.3:37.7	24.6	+4.0
3	(*S*)-**2c**	27.1:72.9	–45.8	–19.2
4	(*S*)-**2d**	71.2:28.8	42.4	+10.3
5	(*S*)-**2e**	79.4:20.6	58.8	+14.0
6	(*S*)-**2f**	28.8:71.2	–42.4	–12.5
7	(*S*)-**2g**	26.5:73.5	–47.0	–21.6
8	(*R*)-**2h**	84.4:15.6	68.8	+32.8
9[c]	(*S*)-**2i**	24.8:75.2	–50.4	–17.7
10[c]	(*S*)-**2j**	44.0:56.0	–12.0	–7.5
11[c]	(*S*)-**2k**	42.4:57.6	–15.2	–5.9
12[c]	(*S*)-**2l**	47.2:52.8	–5.6	–1.8
13[c]	(*S*)-**2m**	69.0:31.0	38.0	+20.4
14[c]	(*S*)-**2n**	77.3:22.7	54.6	+22.2

[a] Excess of *P* conformer (%) = ([*P*]–[*M*])/([*P*]+[*M*])×100, where [*P*] and [*M*] are the amounts of *P* and *M* conformers calculated by B3LYP/6-31G*. [b] A_{CD} value: $A_{CD} = \Delta\varepsilon_1 - \Delta\varepsilon_2$, where $\Delta\varepsilon_1$ and $\Delta\varepsilon_2$ are intensities of first and second Cotton effects, respectively. [c] Kuwahara, S.; Nakamura, M.; Yamaguchi, A.; Ikeda, M.; Habata, Y. *Org. Lett.* **2013**, 15, 5738–5741.

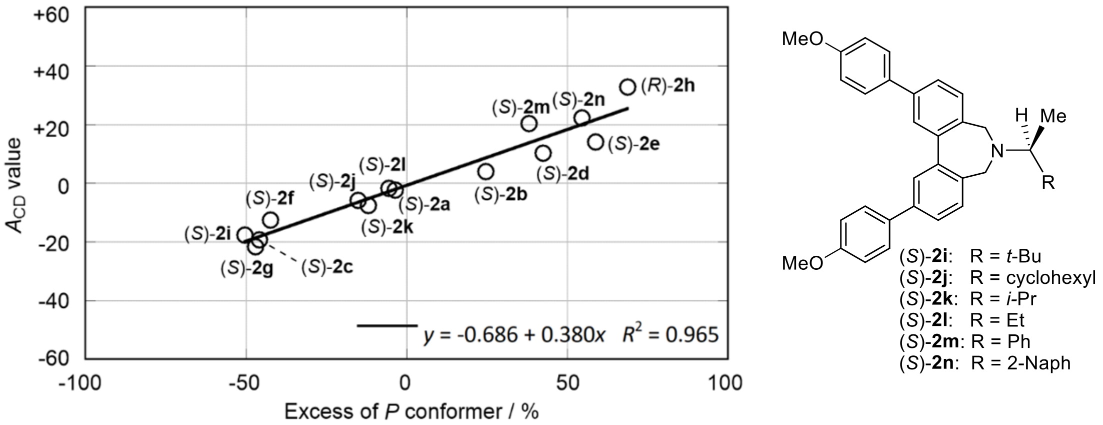

Figure S28. The relationship between the A_{CD} values and excess of *P* conformer. Excess of *P* conformer (%) = ([*P*]–[*M*])/([*P*]+[*M*])×100, where [*M*] and [*P*] are the amounts of *P* and *M* conformers calculated by B3LYP/6-31G*, respectively.

X-ray structure determination

Crystals of (*S*)-**2b** was mounted on the top of a glass fiber, and the data collection was carried out on a Bruker SMART diffractometer equipped with a CCD area detector at 120 K. The data were corrected for Lorentz and polarization effects, and absorption corrections were applied with the *SADABS* probram [3]. The structure was solved by direct methods and subsequent difference Fourier syntheses using the program *SHELXTL* [4]. All non-H atoms were refined anisotropically, and H atoms were placed in calculated positions and thereafter refined with $U_{\mathrm{iso}}(\mathrm{H}) = 1.2U_{\mathrm{eq}}(\mathrm{C})$.

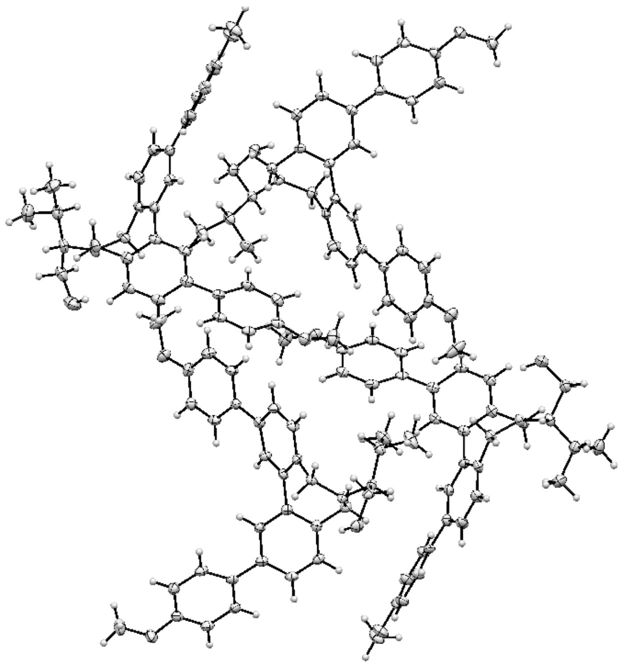

Figure S29. ORTEP The drawing of (*S*)-**2b**.

Table S12. Crystal data and structure refinement for (*S*)-**2b**

Identification code	tri_p1	
Empirical formula	C33 H35 N O3	
Formula weight	493.62	
Temperature	120 K	
Wavelength	0.71073 Å	
Crystal system	Triclinic	
Space group	P1	
Unit cell dimensions	a = 9.5731(4) Å	α = 99.0630(10)°.
	b = 15.1084(7) Å	β = 94.8370(10)°.
	c = 19.7050(9) Å	γ = 108.2140(10)°.
Volume	2646.2(2) Å^3	
Z	4	
Density (calculated)	1.239 Mg/m^3	
Absorption coefficient	0.078 mm^{-1}	
F(000)	1056	
Crystal size	0.32 x 0.26 x 0.12 mm^3	
Theta range for data collection	1.06 to 27.48°.	
Index ranges	-12 <= h <= 12, -19 <= k <= 14, -22 <= l <= 25	
Reflections collected	17127	
Independent reflections	14002 [R(int) = 0.0217]	
Completeness to theta = 27.48°	98.9 %	
Absorption correction	Empirical	
Max. and min. transmission	0.9906 and 0.9754	
Refinement method	Full-matrix least-squares on F^2	
Data / restraints / parameters	14002 / 3 / 1353	
Goodness-of-fit on F^2	1.022	
Final R indices [I > 2sigma(I)]	R_1 = 0.0459, wR_2 = 0.0981	
R indices (all data)	R_1 = 0.0614, wR_2 = 0.1090	
Absolute structure parameter	0.4(9)	
Largest diff. peak and hole	0.229 and -0.255 e.Å^{-3}	

References

[1] Kuwahara, S.; Chamura, R.; Tsuchiya, S.; Ikeda, M.; Habata,Y. *Chem. Commun.* **2013**, *49*, 2186–2188.

[2] Spartan'18, Irvine, CA; Wavefunction, 2018.

[3] Sheldrick, G. M. *Program for absorption correction of area detector frames*; Madison, WI; Bruker AXS, Inc., 1996.

[4] *SHELXTL*, *version 5.1*; Madison, WI; Bruker AXS, Inc., 1997.
